# Genetically Engineered iPSC-Derived FTDP-17 *MAPT* Neurons Display Mutation-Specific Neurodegenerative and Neurodevelopmental Phenotypes

**DOI:** 10.1016/j.stemcr.2019.07.007

**Published:** 2019-08-13

**Authors:** An Verheyen, Annick Diels, Joke Reumers, Kirsten Van Hoorde, Ilse Van den Wyngaert, Constantin van Outryve d’Ydewalle, An De Bondt, Jacobine Kuijlaars, Louis De Muynck, Ronald De Hoogt, Alexis Bretteville, Steffen Jaensch, Arjan Buist, Alfredo Cabrera-Socorro, Selina Wray, Andreas Ebneth, Peter Roevens, Ines Royaux, Pieter J. Peeters

## Main Text

(Stem Cell Reports *11*, 363–379; August 14, 2018)

In the originally published version of our manuscript, we noticed a mistake in the labeling of the actin blots in Figure S6C. The supernatant actin blot was mistakenly assigned to the pellet actin part of the figure and vice versa.

To address this, we have now correctly assigned the actin blots to their respective supernatant and pellet. The corrected panel appears both below and in our supplemental information online. We apologize for the oversight and for any resulting confusion.

**Figure:**
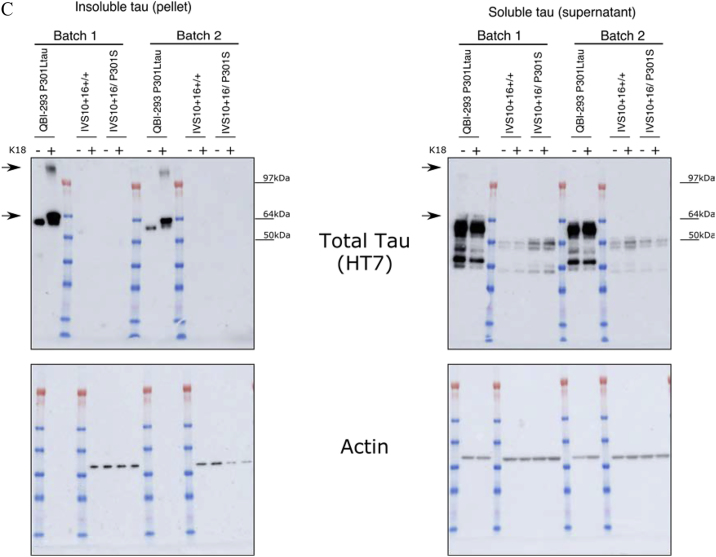
Figure S6C. Seeding-Potent Wild-Type K18 Does Not Induce Aggregation in Single Mutant IVS10+16 Neurons and P301L-K18 Does Not Induce Insoluble Tau (corrected)

**Figure:**
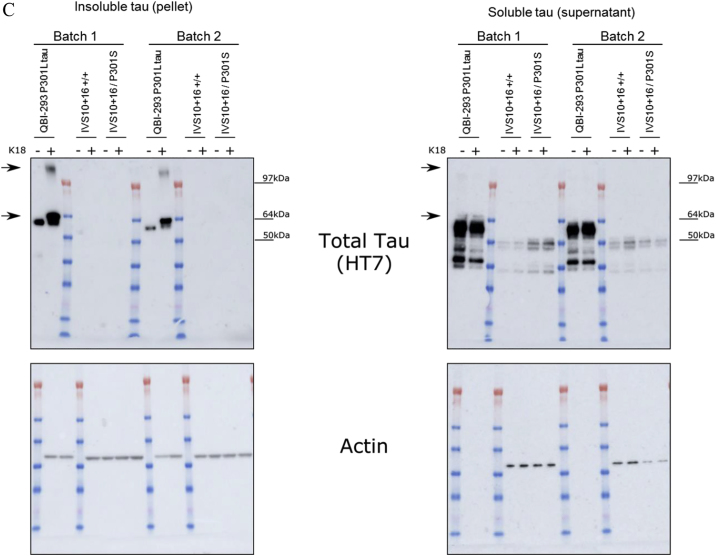
Figure S6C. Seeding-Potent Wild-Type K18 Does Not Induce Aggregation in Single Mutant IVS10+16 Neurons and P301L-K18 Does Not Induce Insoluble Tau (original)

