# Supplementary material for: Genetically Engineered iPSC-Derived FTDP-17 MAPT Neurons Display Mutation-Specific Neurodegenerative and Neurodevelopmental Phenotypes
Source: Stem Cell Reports. 2018 Jul 26;11(2):363–79. doi: 10.1016/j.stemcr.2018.06.022 (PMC6093179; doi:10.1016/j.stemcr.2018.06.022)
Supplement: Document S1. Supplemental Experimental Procedures, Figures S1–S6, and Tables S1–S6 [file mmc1.pdf]

Stem Cell Reports, Volume *11*

Supplemental Information

Genetically Engineered iPSC-Derived FTDP-17 *MAPT* Neurons Display Mutation-Specific Neurodegenerative and Neurodevelopmental Phenotypes

An Verheyen, Annick Diels, Joke Reumers, Kirsten Van Hoorde, Ilse Van den Wyngaert, Constantin van Outryve d'Ydewalle, An De Bondt, Jacobine Kuijlaars, Louis De Muynck, Ronald De Hoogt, Alexis Bretteville, Steffen Jaensch, Arjan Buist, Alfredo Cabrera-Socorro, Selina Wray, Andreas Ebneth, Peter Roevens, Ines Royaux, and Pieter J. Peeters

Supplemental Figures

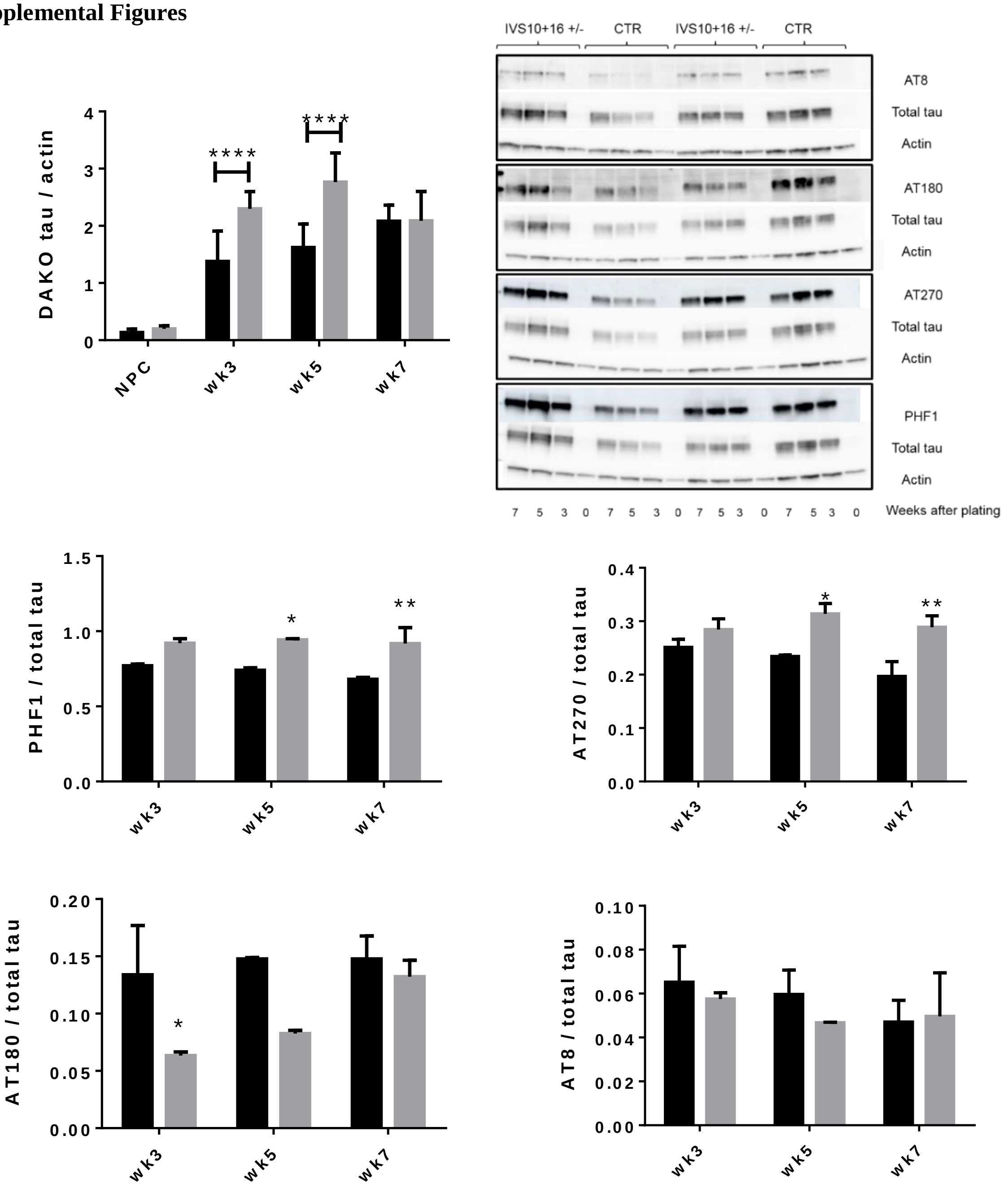

Figure S1: Western blot time course of total tau and phosphorylated tau on control and IVS10+16 +/- neurons. Related to Figure 2. Western blot for total tau and phospho-tau (DAKO total tau, PHF1, AT8, AT270 and AT180; all 1μg/ml). Duplo's from 2 independent differentiations are shown. Phospho tau blots were re-probed with total tau antibodies for quantification. Total tau levels are increased over time in control and mutant neurons ($P<0.0001$, n=4, NPC *versus* wk3 (DIV51), wk5 (DIV65) and wk7 (DIV80) after plating for both control and IVS10+16 cells), with also more total tau in neurons carrying the IVS10+16 mutation at 3 and 5 weeks after plating (n=4; $P<0.0001$). There is an increased phosphorylation at tau Ser396/Ser404 (PHF1) and Thr181 (AT270) in IVS10+16 carrying neurons 5 and 7 weeks after plating while there is less phosphorylation at Thr231 (AT180) 3 weeks after plating; *$P<0.05$, **$P<0.01$ and ****$P<0.0001$ (2-way ANOVA).

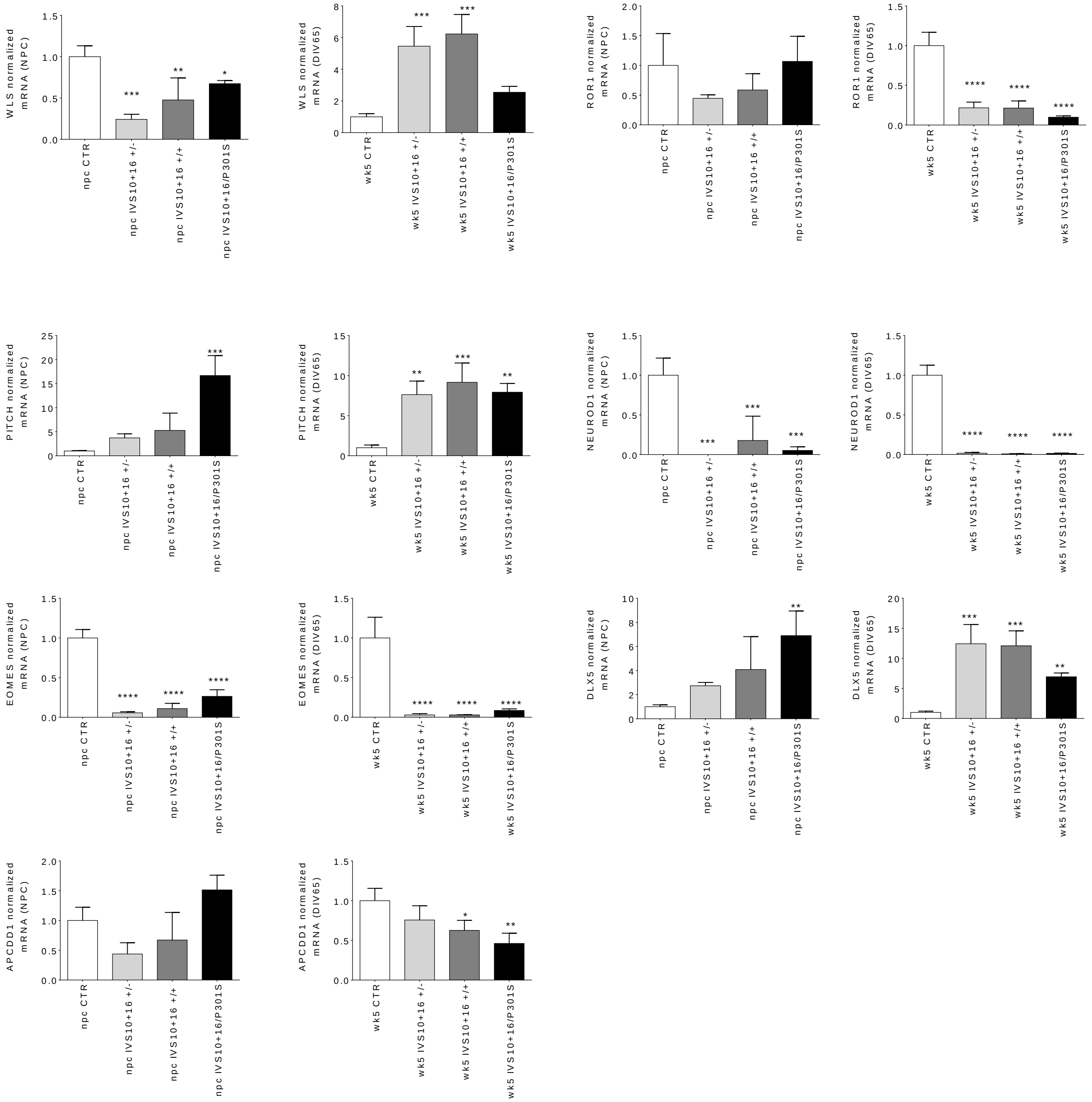

Figure S2: RT-qPCR confirmation of selected genes in control and mutant NPCs and neurons. Related to Figure 3 and Tables S1 and S2. Cells were lysed at DIV31 (NPC) or DIV65 (week 5). *P<0.05, **P<0.01, ***P<0.001 and ****P<0.0001 (1-way ANOVA, n=3).

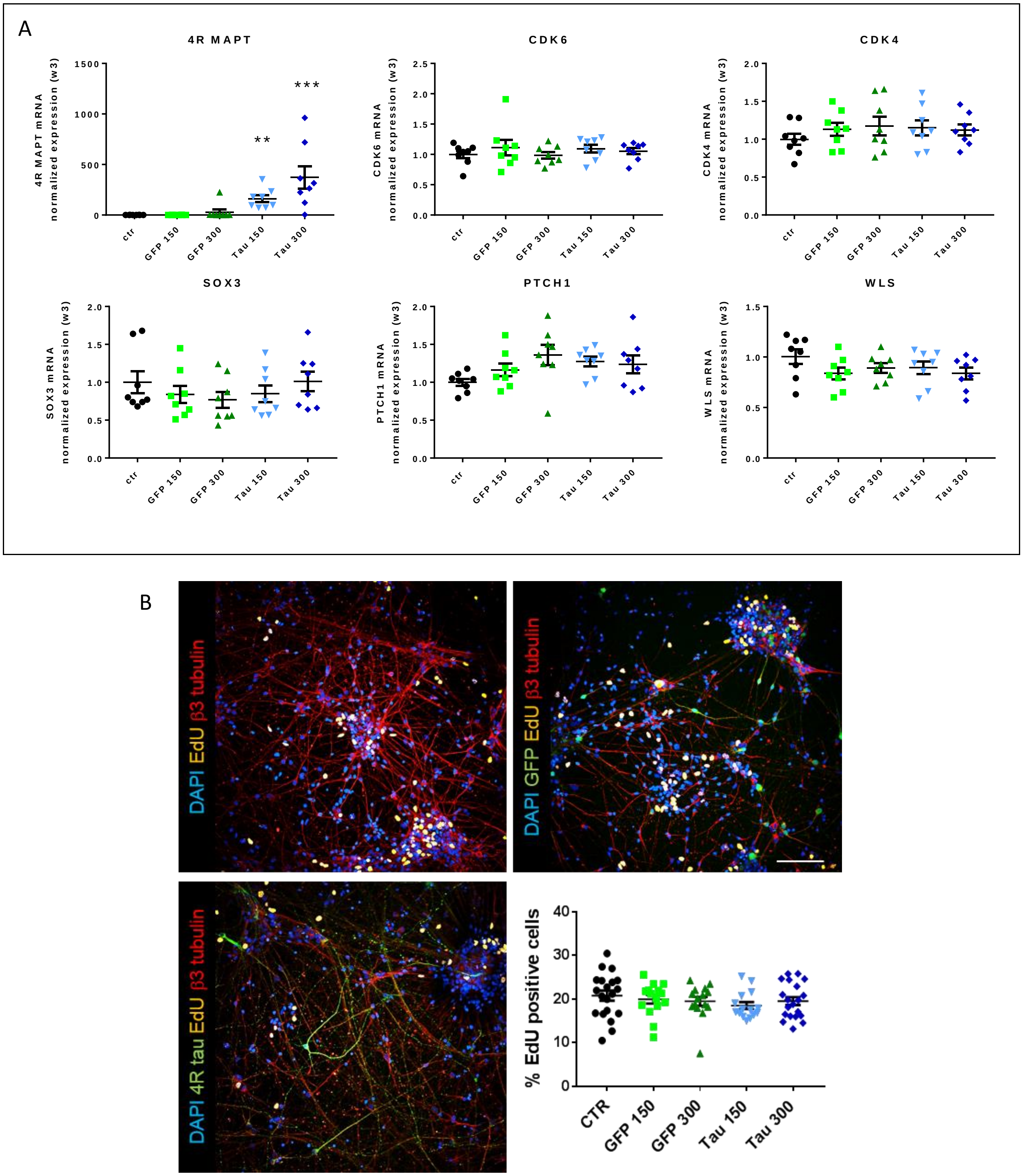

Figure S3: AAV6 induced 4R tau overexpression in control cortical neurons does not recapitulate IVS10+16-induced phenotypes. Related to Figures 3-4 and Figure S2 (A) RT-qPCR on selected genes from cell cycle and SHH/WNT pathways (4R MAPT; $P<0.0001$, $n=3$ and for all other genes $P=NS$, $n=3$). MOI of 150 or 300 was used. The wild type 2N4R tau isoform was used to overexpress 4R tau $^{**}P<0.01$ and $^{***}P<0.001$ (Kruskal Wallis test for 4R MAPT and 1-way ANOVA for all other genes). (B) EdU staining 3 weeks after transduction with AAV ($P=NS$, $n=3$, Kruskal-Wallis test). Representative pictures are shown of control, 4R tau-transduced or GFP-transduced neurons. Scale bar represents 100µm.

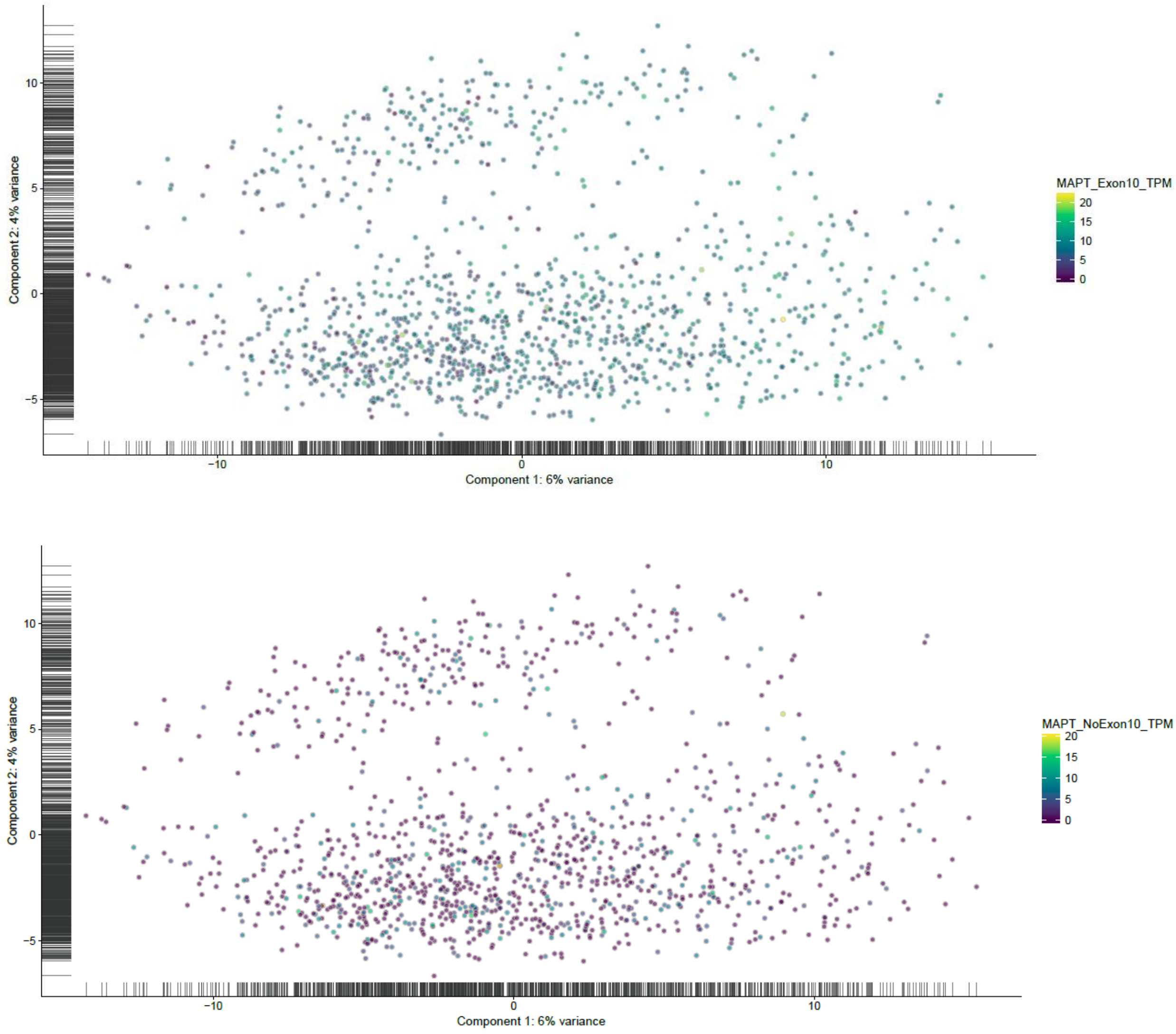

Figure S4: Heterogeneous expression of *MAPT* with and without exon 10 after single cell RNA sequencing of human cerebral cortex tissue. Related to Figure 5. Color mapping of *MAPT* with exon 10 (MAPT_exon10) and *MAPT* without exon 10 (MAPT_no_ex10) reveals a heterogeneous but overall higher expression profile of MAPT_ex10 than MAPT_no_exon10, without clustering of cell populations.

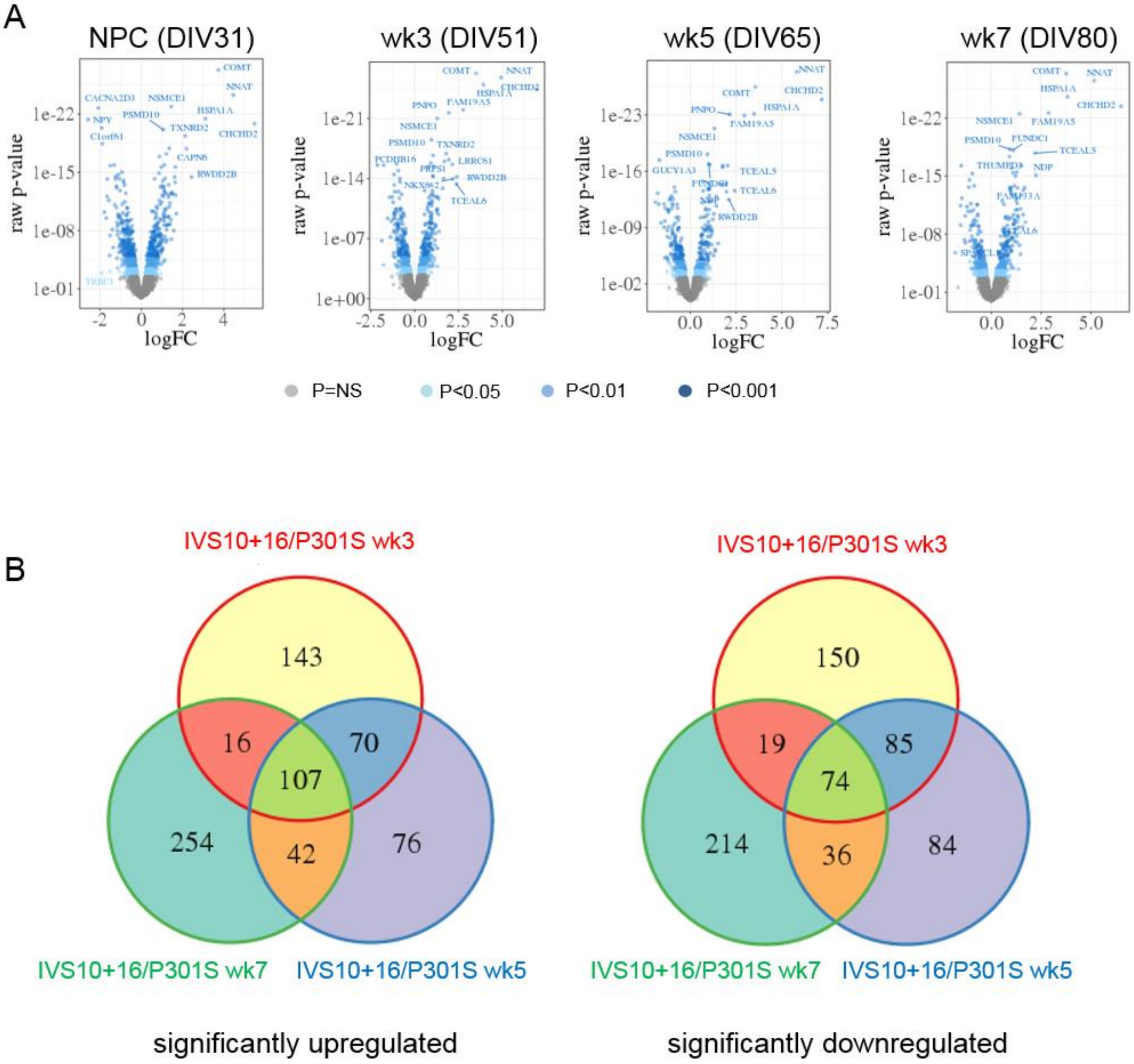

Figure S5: Microarray analysis on IVS10+16 / P301S versus IVS10+16 neurons at different time points. Related to Figure 7. (A) Volcano plots on time points NPC, week 3, week 5 and week 7 after plating. The top genes with the highest significance and/or highest fold change are highlighted. (B) Venn diagrams showing overlapping genes between 3 different neuronal timepoints (3, 5 and 7 weeks after plating) when comparing IVS10+16/P301S neurons to their respective controls (IVS10+16 +/+). 107 genes are significantly upregulated and 74 genes are significantly downregulated at all timepoints.

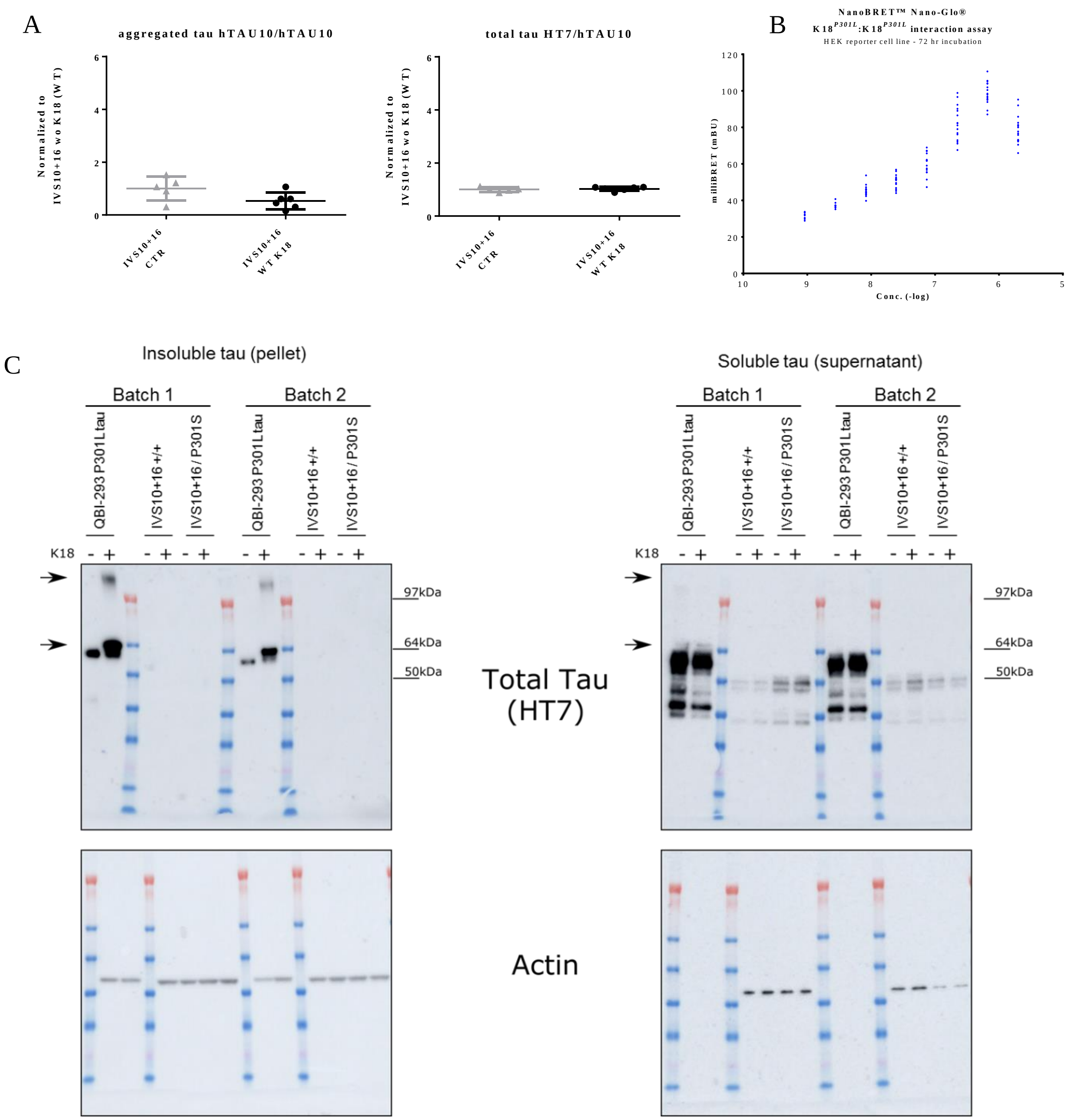

Figure S6: Seeding-potent wild type K18 does not induce aggregation in single mutant IVS10+16 neurons and P301L-K18 does not induce insoluble tau. Related to Figure 7. (A) AlphaLISA for aggregated tau (hTAU10/hTAU10) and total tau shows no difference in signal after addition of wild type K18 seeds (n=3 experiments, P=NS for both, T-test). (B) The NanoBret™Nano-Glo® K18:K18 interaction assay using a HEK reporter cell line (with overexpression of P301L tau) shows that wild type K18 induces a robust, concentration-dependent BRET signal corresponding to tau aggregation after 72 hours of K18-WT incubation. (C) Sarkosyl extraction of insoluble tau from iPSCs derived neurons, with and without K18P301L (lysis 7 weeks after seeding)**.** Equal amounts of protein from iPSC neurons were subjected to Sarkosyl extraction of insoluble material. Insoluble pellets and supernatants were then immunoblotted for total human tau antibody (HT7). Actin immunoblotting of supernatant is used as loading control. Two separate extractions are represented. Lysate of QBI293 cells transiently transfected with P301L tau and seeded with K18 fibrils (dilution 1/30 compared to the other samples), is used in each extraction batch as a positive control of successful extraction of insoluble tau.

Table S1: Differential expression of genes related to neuronal subtypes and cell cycle regulation due to IVS10+16 mutation. Related to Figures 3 and 4.

**Significantly downregulated forebrain genes (logFC) IVS10+16 *vs* control**	**Significantly upregulated interneuron/basal ganglia/limbic genes (logFC) IVS10+16 *vs* control**	**Significantly different transcription factors/ cell cycle regulators (logFC) IVS10+16 *vs* control**
*EOMES (-5.00) *** *	*ISL1 (4.21) **** *	*TCF3 (-0.39) ****
*TBR1 (-3.78) **** *	*RELN (4.07) **** *	*TCF4 (-0.87) **** *
*SATB2 (-1.85) **** *	*DLX6 (3.89) **** *	*HEY2 (-1.22) **** *
*NEUROD1 (-4.63) **** *	*DLX2 (2.80) **** *	*SOX3 (-2.64) **** *
*NEUROD2 (-5.46) **** *	*DLX1 (2.72) **** *	*ZFPM2 (-4.63) **** *
*NEUROD6 (-7.25) **** *	*DLX5 (3.05) **** *	*CDK2 (-0.70) **** *
*EMX1 (-2.61) **** *	*GAD2 (2.45) **** *	*CDK4 (-1.03) **** *
*EMX2 (-2.95) **** *	*GAD1 (2.31) **** *	*CDK6 (2.64) **** *
*SLC17A7 (-3.86) **** *	*SLC32A1 (3.29) **** *	*CDK17 (0.63) **** *
*SLC17A6 (-4.22) **** *	*LHX6 (2.51) **** *	*CDK18 (-0.58) **** *
*LHX2 (-2.28) **** *	*LHX8 (3.64) **** *	*CDK19 (0.48) ***
*FEZF2 (-6.02) **** *	*GSX2 (2.30) **** *	*CDK20 (0.71) ****
*NEUROG2 (-5.69) **** *	*LSAMP (1.67) **** *	*p18 CDKN2C (1.06) **** *
*NEUROG1 (-2.25) **** *	*TH (1.17) **** *	*p19 CDKN2D (0.62) **** *
*LEF1 (-2.77) **** *	*DRD1 (1.48) **** *	*p21 CDKN1A (-1.63) **** *
*HES1 (-2.26) **** *	*DRD2 (0.92) **** *	*p27 CDKN1B (-0.46) ***

Table S1: List of differentially expressed genes after microarray on ZFN monoallelic *MAPT* IVS10+16 neurons five weeks after final plating and compared to the isogenic control. A selection of genes related to forebrain, interneuron/basal ganglia, limbic system and transcription factors/cell cycle regulators is shown. LogFC (fold changes) are shown. **P<0.01; ***P<0.001 and ****P<0.0001. See also Figures 3 and 4.

Table S2: Differential expression of genes related to WNT and SHH signaling at NPC and neuronal stage due to IVS10+16 mutation. Related to Figure 6.

WNT/SHH signaling significant GENES	Time point	Sigma IVS10+16 DIV65 (logFC)	Patient 1 IVS10+16 DIV65 (logFC)	Patient 2 IVS10+16 DIV65 (logFC)
WLS	***DIV65***<br>***NPC***	↑ (2.05) ****<br>↓ (-1.88) ****	↑ (1.21) ****<br>↓ (-2.53) ****	↑ (2.07) ****
WNT4	***DIV65***	↑ (0.51) ****	↑ (0.84) ****	↑ (0.27) *
WNT7A	***DIV65***<br>***NPC***	-<br>↑ (0.78) ****	-<br>-	↑ (0.60) ***<br>↑ (1.38) ****
WNT7B	***DIV65***<br>***NPC***	↓ (-0.30) ***<br>↓ (-0.35) ***	↓ (-0.37) ****<br>↓ (-0.44) ****	↓ (-0.30) ***<br>↓ (-0.34) ***
WNT5B	***DIV65***<br>***NPC***	↓ (-0.34) *<br>-	↓ (-0.54) ***<br>↑ (0.73) ***	↓ (-0.72) ****<br>↑ (0.76) ***
GSK3A	***DIV65***<br>***NPC***	↑ (0.20) *<br>-	↑ (0.21) **<br>↓ (-0.21) *	-<br>-
GSK3B	***DIV65***	↑ (0.27) ***	↑ (0.52) ****	↑ (0.66) ****
AXIN2	***DIV65***	↑ (0.29) **	↑ (0.46) ****	↑ (0.28) **
TCF7L2	***DIV65***<br>***NPC***	-<br>↑ (0.56) ****	↑ (0.20) *<br>↑ (0.40) ***	↑ (0.36) ***<br>-
TCF7L1	***DIV65***<br>***NPC***	↓ (-0.87) ****<br>-	↓ (-0.72) ****<br>↑ (0.43) *	↓ (-0.74) ****<br>-
FZD8	***DIV65***<br>***NPC***	↓ (-0.62) ****<br>-	↓ (-0.46) ****<br>↑ (0.38) ***	↓ (-0.67) ****<br>-
FZD7	***DIV65***<br>***NPC***	↓ (-1.55) ****<br>↓ (-0.85) ****	↓ (-1.97) ****<br>↓ (-1.01) ****	↓ (-1.89) ****<br>↑ (0.69) ****
FZD3	***DIV65***<br>***NPC***	↑ (0.73) ****	↑ (1.30) ****<br>↑ (0.45) ***	↑ (1.06) ****
FZD5	***DIV65***<br>***NPC***	↑ (0.28) *<br>↑ (1.65) ****	↑ (0.93) ****<br>↑ (2.28) ****	-<br>↑ (1.22) ****
FZD2	***DIV65***<br>***NPC***	↓ (-0.81) ****	↓ (-0.66) ****<br>↑ (0.48) ***	↓ (-0.29) *
FZD9	***DIV65***<br>***NPC***	↓ (-0.36) *	-	↓ (-0.42) **<br>↑ (0.51) **
FZD6	***DIV65***<br>***NPC***	↓ (-0.81) ****<br>↓ (-0.46) **	-	↓ (-0.33) **
NIN	***DIV65***<br>***NPC***	↑ (0.50) ****	↑ (0.72) ****<br>↑ (0.49) ****	↑ (1.29) ****
SRFP1	***DIV65***<br>***NPC***	↓ (-0.43) **<br>↑ (0.30) *	↓ (-0.36) **<br>↑ (0.90) ****	-<br>↑ (0.54) ***
LRP2	***DIV65***<br>***NPC***	-<br>↑ (0.90) ****	-<br>↑ (0.92) ****	↑ (0.29) *<br>↑ (1.72) ****
PTCH1	***DIV65***<br>***NPC***	↑ (1.17) ****<br>↑ (1.25) ****	↑ (2.24) ****<br>↑ (1.00) ****	↑ (1.73) ****<br>↑ (1.15) ****
PTCHD1	***DIV65***<br>***NPC***	↑ (1.50) ****<br>↑ (2.14) ****	↑ (2.63) ****<br>↑ (2.33) ****	↑ (1.25) ****<br>↑ (1.55) ****
CEP76	***DIV65***<br>***NPC***	↓ (-0.46) ***<br>↓ (-0.42) **	↓ (-0.62) ****<br>↓ (-0.65) ****	↓ (-0.66) ****<br>↓ (-0.61) ****
CAV1	***DIV65***<br>***NPC***	↓ (-1.25) ****<br>↓ (-0.52) **	↓ (-1.33) ****<br>↓ (-0.42) **	↓ (-1.30) ****<br>↓ (-0.59) ***
APCDD1	***DIV65***<br>***NPC***	↓ (-0.56) ***<br>↓ (-0.94) ****	↓ (-0.36) *<br>↓ (-0.23) ****	-<br>↓ (-0.51) **
ROR1	***DIV65***<br>***NPC***	↓ (-0.72) ****<br>↓ (-0.84) ****	↓ (-0.76) ****<br>↓ (-0.72) ****	↓ (-0.44) ***<br>↓ (-0.47) ***

Table S2: Differentially expressed genes from the *WNT* and *SHH* signaling pathways after microarray on ZFN (Sigma) and patient-derived IVS10+16 NPCs and neurons (DIV65, in blue color) compared to the Sigma parental control. LogFC (fold changes) are shown for all significantly different genes with P<0.05. *P<0.05, **P<0.01, ***P<0.001 and ****P<0.0001. See also Figure 6.

Table S3: Differential expression of genes related to MAPT exon 10 splicing mediators due to IVS10+16 mutation. Related to Figure 6.

MAPT exon 10 splicing mediator GENES	Sigma IVS10+16 (logFC)	Patient 1 IVS10+16 (logFC)	Patient 2 IVS10+16 (logFC)
CELF3	↑ (0.36) *** NPC	↑ (0.27) ** NPC	↑ (0.32) ** NPC
CELF4	↓ (-0.52) *** NPC<br>↑ (0.56) *** wk5	↑ (0.55) *** NPC<br>↑ (1.25) **** wk5	↑ (1.03) **** NPC<br>↑ (0.89) **** wk5
NOVA1	↑ (0.64) **** NPC<br>↑ (1.33) **** wk5	↑ (0.90) **** NPC<br>↑ (1.92) **** wk5	-<br>↑ (1.76) **** wk5
SWAP70	-<br>↓ (-0.46) *** wk5	↑ (0.37) * NPC<br>↓ (-0.55) *** wk5	-<br>↓ (-0.42) ** wk5
SRSF3	↑ (0.27) ** wk5	-	↑ (0.38) **** wk5
SRSF1	↑ (0.39) **** wk5	-	↑ (0.62) **** wk5
SRSF4	↓ (-0.25) ** wk5	↓ (-0.30) *** wk5	-
SRSF6	↑ (0.78) **** wk5	↑ (0.85) **** wk5	↑ (1.26) **** wk5
DDX5	↑ (0.19) ** wk5	↑ (0.21) ** wk5	↑ (0.42) **** wk5
PTBP1	↓ (-0.58) **** NPC<br>↓ (-0.43) **** wk5	↓ (-0.34) **** wk5	↑ (0.15) * wk5
PRKACA	-<br>↑ (0.42) *** wk5	-<br>↑ (0.50) **** wk5	↑ (0.24) * NPC<br>↑ (0.30) ** wk5
DYRK1A	↑ (0.22) ** wk5	↑ (0.26) ** wk5	↑ (0.40) **** wk5
CLK2	↓ (-0.51) **** wk5	↓ (-0.30) ** wk5	-

Table S3: Differentially expressed genes from the *MAPT* exon 10 splicing machinery after microarray on ZFN (Sigma) and patient-derived IVS10+16 NPCs and neurons compared to the Sigma parental control. LogFC (fold changes) are shown for all significantly different genes with P<0.05. *P<0.05, **P<0.01, ***P<0.001 and ****P<0.0001. See also Figure 6.

Table S4: Differential expression of genes related to AD, PD and FTD due to IVS10+16 mutation. Related to Figure 6.

AD, PD and FTD related GENES	Sigma IVS10+16 (logFC)	Patient 1 IVS10+16 (logFC)	Patient 2 IVS10+16 (logFC)
APOE	(-1.50) **** wk5	(-1.48) **** wk5	(-1.73) **** wk5
BACE1	(0.35) *** wk5	(0.44) **** wk5	-
MAPT	(1.04) **** wk5	(1.37) **** wk5	(0.70) **** wk5
FUS	(0.24) *** wk5	-	(0.27) **** wk5
TARDBP	(0.22) * wk5	(0.28) ** wk5	(0.58) **** wk5
GRN	(-0.29) ** NPC	(-0.41) *** NPC	(-0.31) *** NPC<br>(-0.25) * wk5
CHCHD2	(-0.55) **** NPC<br>(-0.95) **** wk5	(-0.42) **** NPC<br>(-0.95) **** wk5	(-0.22) ** NPC<br>(-0.72) **** wk5
GAK	(0.47) ** wk5	(0.83) **** wk5	(0.56) *** wk5

Table S4: AD, PD and FTD-related differentially expressed genes after microarray on ZFN (Sigma) and patient-derived IVS10+16 NPCs and neurons compared to the Sigma parental control. LogFC (fold changes) are shown for all significantly different genes with P<0.05. *P<0.05, **P<0.01, ***P<0.001 and ****P<0.0001. See also Figure 6.

Table S5: Differential expression of genes related to calcium signaling due to P301S mutation. Related to Figure 7.

Significantly different genes potentially related with phenotypes	IVS10+16 / P301S DIV65 / DIV80 (logFC)	P values DIV65 / DIV80
CACNA2D3	↓ (-1.04) / (-0.67)	**** / ****
SLC8A1	↓ (-0.54)	**** (DIV65)
S100A16	↓ (-0.58) / ↓ (-0.60)	*** / ***
SMOC1	↓ (-0.74) / ↓ (-0.61)	** / *
CABYR	↑ (0.57)	** (DIV65)
S100A10	↑ (0.53) / ↑ (0.56)	* / *
NRXN3	↓ (-0.70) / ↓ (-0.65)	**** / ***
PRKCB	↓ (-0.38)	* (DIV65)

Table S5: List of differentially expressed genes after microarray analysis on P301S/IVS10+16 five to seven weeks after final plating and compared to its respective control (IVS10+16 +/+). Calcium signaling-related genes have been selected with a potential link to the observed phenotype. LogFC (fold changes) are shown. *P<0.05; **P<0.01; ***P<0.001; ****P<0.0001. See also Figure 7.

Supplemental experimental procedure

Human iPSC culture and differentiation into cortical neurons

Human iPSCs were cultured feeder-free and fed daily with fresh mTeSR™1 medium (Stem Cell Technologies). Cells were passaged with EDTA (Gibco) at confluency. Differentiation into neural progenitor cells (NPCs) and cortical neurons was performed using an adapted dual SMAD inhibition protocol (Kuijlaars et al., 2016, Shi et al., 2012). The only difference with the original protocol (Shi et al., 2012) implies the addition of BDNF, GDNF (both 10ng/ml) and cAMP (100μM) to the neural maintenance medium, leading to a mixture of both glutamatergic and GABAergic cortical neurons instead of purely glutamatergic neurons. For functional calcium experiments, neurons were co-cultured with human primary astrocytes (Kuijlaars et al., 2016) (ScienCell™) that were cultured and passaged per manufacturer's instructions (in human astrocyte medium, ScienCell™). All control and ZFN gene edited lines are deposited in EBiSC (https://cells.ebisc.org) as SIGi001-A (parental control, female), SIGi001-A-9 (IVS10+16 bi-allelic + P301S bi-allelic), SIGi001-A-12 (IVS10+16 bi-allelic) and SIGi001-A-13 (IVS10+16 mono-allelic). The iPSC lines from two female patients carrying the IVS10+16 (mono-allelic) mutation (patient 1 and patient 2) have been described (Sposito et al., 2015). Both patients were pre-symptomatic at the time of biopsy.

Seamless introduction of point mutations by gene editing using ZFN technology

Gene editing was performed by Sigma Aldrich on the control human iPSC line (cat#IPSC0028). Briefly, low passage number feeder free cultured hiPSCs were transfected with *in vitro* transcribed ZFN and single strand DNA oligo (ssODN) donor template by Amaxa nucleofection. The ZFN targets sequence CCT TCA CAC GTC CCA TGC GCC GTG CTG TGG CTT GAA TTA TTAG in intron 10 (underlined: ZFN binding sites). The 2 ssODNs used in this study were 102-nt long and contained the IVS10+16 SNP (C>T underlined) alone or in combination with the P301S point mutation (C>T in bold) (ssODN sequence= GTG GCT CAA AGG ATA ATA TCA AAC ACG TC**T** CGG GAG GCG GCA GTG TGA GTA CCT TCA CAT GTC CCA TGC GCC GTG CTG TGG CTT GAA TTA TTA GGA AGT GGT. ZFN activity was confirmed on genomic DNA from nucleofected cell pool in the mismatch cleavage assay. Cells were plated in pools and screened by Restriction Fragment Length Polymorphism (RFLP) using *Pci*I restriction enzyme for the presence of the IVS10+16 (C>T) point mutation and *Bsm*B1 for the presence of the p.P301S (C>T) point mutation. Selected pools were then single-cell plated and correctly edited clones identified by Sanger sequencing. Clonal populations containing either mono- or bi-allelic modifications for P301S alone or in combination with IVS10+16 were characterized for pluripotency (flow cytometry) and genomic stability (G banding).

RNA extraction and RTqPCR

Cells were lysed with RLT buffer (Qiagen) supplemented with 1% β-mercaptoethanol. RNA extraction was done using the RNeasy mini kit (Qiagen) followed by cDNA preparation using SuperScript® III (Life Technologies). For RTqPCR, the following Taqman assays to detect total MAPT and housekeeping genes were purchased at Life technologies total MAPT (Hs00902194_m1), PGK1 (Hs99999906_m1), HMBS (Hs00609297_m1) and PPIB (Hs00168719_m1). SOX3, CDK6, CDK4, WLS, ROR1, PITCH, NEUROD1, EOMES, DLX5 and APCDD1 Taqman assays were purchased at IDT. The assays to specifically detect the MAPT variants with or without exon 10 were custom designed and ordered at IDT: TaqMan assay MAPT_ 3R_exon 9_11 (Fwd primer: GCT CCA CTG AGA ACC TGA AG, Rev primer: CCT AAT GAG CCA CAC TTG GA, Probe 56-FAM/AG ACT ATT T/Zen/G CAC CTT CCC GCC TC/3IABkFQ) and TaqMan assay MAPT_4R_Exon 9_10 (Fwd primer: GCT CCA CTG AGA ACC TGA AG, Rev: TTG AGC CAC ACT TGG ACT G, Probe 56-FAM/AA TTA TCT G/Zen/C ACC TTC CCG CCT CC/3IABkFQ. Normalization was done using the geNorm method ((Vandesompele et al., 2002)) using reference genes (B2M, HMBS, PGK1, PPIB, TFRC). A normalization factor was computed as the geometric averaging of the gene expression values of the most stable reference genes. The gene expression values were divided by this normalization factor. A second normalization was performed to the parental control at NPC stage.

Microarray analysis

For microarray analysis, RNA was extracted with the RNeasy 96 kit (cat# 74181 Qiagen). All microarray-related steps for target preparation, including the amplification of total RNA and labeling, were carried out as described in the GeneChip®3' IVT Express Kit User Manual (Affymetrix 2004). Biotin-labeled target samples were hybridized to the GeneChip® HG-U219 containing probes for over 18k genes. Target hybridization was processed on the GeneTitan® Instrument according to the instructions provided in the User Guide for Expression Array Plates (P/N 702933). Images were analyzed using the GeneChip® Command Console Software (AGCC) (Affymetrix). All microarray data were processed using the statistical computing R-program (R version 3.1.1 (Team, 2015)) as well as Bioconductor tools (Gentleman et al., 2004). The gene expression values were normalized using Robust Multi-array Average (RMA)

(Irizarry et al., 2003). Grouping of the individual probes into gene-specific probe sets was performed based on Entrez Gene using the metadata package hgu219hsentrezg (version 20.0.0) (Dai et al., 2005).

Single cell data analysis

Single-nucleus RNA sequencing data originating from the cerebral cortex of a postmortem brain (Lake et al., 2016) were downloaded from the Sequence Read Archive (accession SRP052546). Transcript-level quantification was performed with kallisto (Bray et al., 2016) using Ensembl release 89 as a reference transcriptome, and further processed using the R Bioconductor package scater (McCarthy et al., 2017). Gene-level summarization was performed by grouping all transcripts in a given gene, except for the MAPT gene, where grouping was performed using transcripts either including or excluding exon 10.

Live Cell Calcium Imaging

Cells were loaded with 1 µM Fluo-4-AM (Thermo Fisher Scientific) in D-PBS containing calcium and magnesium (cat# D8662; Sigma) with addition of 10mM glucose. Cultures were incubated at 37°C and 5% CO2 for 30 minutes and then imaged with an inverted confocal laser scanning microscope (Axiovert 100M Carl Zeiss, combined with Zeiss LSM510 software) using a Plan-NEOFLUAR 20x objective lens (NA 0.50). 250 frames (61 frames per minute) were recorded per well followed by 30 µM glutamate addition (50 frames) to distinguish neurons from non-neuronal cells (Pickering et al., 2008). Traces of non-neuronal cells, showing only a transient increase in fluorescence intensity upon glutamate addition, were discarded. A custom-made MATLAB script (based on (Cornelissen et al., 2013)) was used to analyze live cell calcium traces and to derive various parameters reflecting characteristics of neuronal activity. Fluorescence traces were normalized to the initial fluorescence intensity (F/F0) and the average calcium burst frequency was calculated for the active cells. Active cells were defined as cells showing at least one peak (i.e. calcium burst) in the fluorescence signal.

Western Blot

Cells were washed gently with PBS and lysed in RIPA buffer (Gibco) supplemented with protease and phosphatase inhibitors (HALT®; Invitrogen). To detect separate 3R and 4R tau isoforms, some of the samples were dephosphorylated with lambda phosphatase (New England Biolabs) according to the manufacturer's protocol after acetone precipitation, to obtain a maximum yield of proteins. Protein was loaded on either 4-12% or 10% Criterion Bis-Tris gels (Biorad) and after SDS PAGE, gels were blotted on nitrocellulose and blocked for 1 hour at room temperature (RT) in TBS-0.1% Tween-20 supplemented with 5% milk. The primary rabbit anti-total tau (1□g/ml; DAKO #A0024), mouse anti-RD4 (1/1000; Millipore, 50µg protein loaded), mouse anti-RD3 (1/2000; Millipore, 10 µg protein loaded) antibodies was were incubated overnight at 4^{0}C in blocking buffer. A tau ladder (rPeptide) with all 6 tau isoforms was included to distinguish between 3R and 4R isoforms after dephosphorylation. Detection was done with HRP-labeled secondary antibodies (GE Healthcare) and the West Dura® or West Femto® enhanced chemiluminescence kit (Pierce, Thermo Scientific). Blots were stripped and reprobed with mouse anti-β-actin (1/5000; Sigma) and anti-β3 tubulin (1/1000; Covance) as loading controls.

Preparation of K18 fibrils

Monomeric tau K18-P301L or K18-wild type (40 µM, N and C-terminal myc-tagged) was mixed with 40 µM of heparin, 2mM DTT and 100 mM sodium acetate buffer (pH 7.0) and incubated at 37°C for 48 - 72 hours. Afterwards, the mix was centrifuged (100.000g, 1 hour at 4°C). The supernatant was discarded and the pellet was resuspended in the same final volume of sodium acetate buffer. K18 was freshly sonicated before use (60 cycles of 2 second pulses).

Transduction of NPCs with AAV6-4R tau of AAV6-GFP

Control cortical neural progenitor cells in MW6 plates were transduced with AAV6-syn1-TAU-2N4R (produced by Sebastian Kügler, Dept. of Neurology, University Medicine, Goettingen, Germany) or AAV6-syn1-GFP (produced by SIRION Biotech) at an MOI of 150 or 300 and with final plating of the cells 24 hours later in PLO/laminin coated MW96 plates.

Generation of Tau BRET reporter cell line and Nano BRET™ Nano-Glo® assay

Synthetic fragments of Nano-Luc, an ATP-independent luciferase, or HaLo tag from Promega were cloned in-frame to the C-terminal side of a K18 Tau fragment existing solely of the four MT binding domains with P301L mutation. Both were cloned into the pcDNA5/TO or pcDNA4/TO mammalian expression vector respectively (Invitrogen, Carlsbad, CA, USA). In these vectors, receptor expression is under the control of a CMV promoter and two tetracycline operators, which confers tetracycline-inducible expression on the insert. Hek293 cells were co-transfected

with K18P301L-NanoLuc-pcDNA5/TO, K18P301L-HaloTag-pcDNA4/TO, and pcDNA6/TR expressing the Tet repressor (Invitrogen, Carlsbad, CA, USA) using lipofectAMINE 2000 (Gibo-BRL, Eggenstein, Germany) according to the recommendations of the supplier. Monoclonal cell lines were isolated under Zeocin (200 mg/ml), Hygromycin (200 µg/ml), and Blasticidin (5 mg/ml) in DMEM medium supplemented with 10% heat inactivated fetal calf serum and antibiotics (Life Technologies, Gaithersburg, MD, USA). The same medium was used for cell culturing. Zeocin, Hygromycin, and Blasticidin were left out at least 1 day before any assay. The selected monoclonal line used in the assay showed expression of both constructs in the absence of tetracyclin, therefore the Tet repressor was not blocked before the BRET assay. Wild type K18 fibrils were added to the cell line and incubated for 72 hours, followed by addition of NanoBRET™ Nano-Glo® Substrate and measurement of the signal (donor emission at 460 nm and acceptor emission at 618 nm) within 10 minutes after substrate addition.

Tau insoluble Sarkosyl extraction

Cell pellets were thawed on ice and lysed in RIPA buffer (Sigma # R0278) (Merck, Sigma) supplemented with cOmplete™ (EDTA free) and PhosSTOP™ (Roche) as recommended by the manufacturer. Samples were homogenized on a rocker (IKA Loopster digital) at 4°C. Equal amounts of protein (50-100µg) were aliquoted after BCA protein quantification (Sigma BCA1-1KT) and supplemented with a 20% N-Lauroylsarcosine (Sigma # L9150) solution in RIPA to bring N-Lauroylsarcosine at a final concentration of 1% (w/v). Samples were homogenized on a rocker for 1 hour at room temperature. Samples were then centrifuged at 100,000g for 1 hour at room temperature in a Beckman coulter ultima ultracentrifuge using a TLA-100 rotor and corresponding centrifugation tubes (Beckman, coulter #342303). Supernatants (Soluble fraction) were separated from pellets (insoluble fraction) and kept at -80°C until further analysis. For western Blots analysis, supernatant or pellets were supplemented with LDS (ThermoFisher, Life Technologies, # NP0005) and sample reducing agent (ThermoFisher, Life Technologies, # NP0004) and loaded onto Novex Nupage gels as recommended by the manufacturer. Human Tau monoclonal antibody HT7 (ThermoFischer, MN1000) and actin (Merck, Millipore, # MAB1501) antibodies were used for detection. As a positive control, QBI cells overexpressing 2N4R-TauP301L and seeded with K18P301L were used. The cells were generated by transient transfection with 2N4R-TauP301L-pcDNA4 plasmid in optiMEM using Fugene6 transfection reagent (Promega) according to manufacturer's recommendation. After 24 hours, cells were then either seeded or not with sonicated K18P301L fibrils in a Bioporter (BP609504) sodium acetate buffer (0.1M, pH7) solution and incubated for 3 hours. Cell medium was then replaced with fresh optiMEM supplemented with 20% Foetal bovine serum and 2% Penicillin/streptomycin (Life Technologies) and then cultured for 48 hours prior lysis in RIPA buffer with protease and phosphatase inhibitors as described above.

Table S6: Antibody information. Related to materials and methods.

Antibody	Provider	Catalogue number
rabbit anti tau	DAKO	A0024
mouse anti-actin	Millipore	MAB1501
rabbit anti-VGLUT2	Synaptic systems	135403
rabbit anti-OCT4	Invitrogen	A13998
mouse anti- ISL1	Thermo Fisher	MA5-15516
mouse anti-RD4	Millipore	05-804
mouse anti RD3	Millipore	05-803
mouse anti actin	Millipore	MAB1501
mouse anti-NANOG	Millipore	MABD24
mouse and rabbit anti-TUBB3	Covance<br>(BioLegend)	MMS-435P<br>PRB-435P<br>MRB-435P
rabbit anti-TTF1	Abcam	ab76013
rabbit anti-TBR1	Abcam	ab31940
rat anti-CTIP2	Abcam	ab18465
Click-iT® Plus EdU Alexa Fluor® 594 Imaging kit	Invitrogen	C10639
TUNEL (Roche) kit	Roche/Sigma	11 684 795 910

Supplemental References

J. Kuijlaars, T. Oyelami, A. Diels, J. Rohrbacher, S. Versweyveld, G. Meneghello, M. Tuefferd, P. Verstraelen, J. R. Detrez, M. Verschuuren, et al. Sustained synchronized neuronal network activity in a human astrocyte co-culture system. *Sci Rep,* 6 (2016)**,** 36529.

Y. Shi, P. Kirwan & F. J. Livesey Directed differentiation of human pluripotent stem cells to cerebral cortex neurons and neural networks. *Nat Protoc,* 7 (2012)**,** 1836-46.

T. Sposito, E. Preza, C. J. Mahoney, N. Seto-Salvia, N. S. Ryan, H. R. Morris, C. Arber, M. J. Devine, H. Houlden, T. T. Warner, et al. Developmental regulation of tau splicing is disrupted in stem cell-derived neurons from frontotemporal dementia patients with the 10 + 16 splice-site mutation in MAPT. *Hum Mol Genet,* 24 (2015)**,** 5260-9.

J. Vandesompele, K. De Preter, F. Pattyn, B. Poppe, N. Van Roy, A. De Paepe & F. Speleman Accurate normalization of real-time quantitative RT-PCR data by geometric averaging of multiple internal control genes. *Genome Biol,* 3 (2002)**,** RESEARCH0034.

R. C. Team A language and environment for statistical computing. R Foundation for Statistical Computing, Vienna, Austria. (2015).

R. C. Gentleman, V. J. Carey, D. M. Bates, B. Bolstad, M. Dettling, S. Dudoit, B. Ellis, L. Gautier, Y. Ge, J. Gentry, et al. Bioconductor: open software development for computational biology and bioinformatics. *Genome Biol,* 5 (2004)**,** R80.

R. A. Irizarry, B. Hobbs, F. Collin, Y. D. Beazer-Barclay, K. J. Antonellis, U. Scherf & T. P. Speed Exploration, normalization, and summaries of high density oligonucleotide array probe level data. *Biostatistics,* 4 (2003)**,** 249-64.

M. Dai, P. Wang, A. D. Boyd, G. Kostov, B. Athey, E. G. Jones, W. E. Bunney, R. M. Myers, T. P. Speed, H. Akil, et al. Evolving gene/transcript definitions significantly alter the interpretation of GeneChip data. *Nucleic Acids Res,* 33 (2005)**,** e175.

B. B. Lake, R. Ai, G. E. Kaeser, N. S. Salathia, Y. C. Yung, R. Liu, A. Wildberg, D. Gao, H. L. Fung, S. Chen, et al. Neuronal subtypes and diversity revealed by single-nucleus RNA sequencing of the human brain. *Science,* 352 (2016)**,** 1586-90.

N. L. Bray, H. Pimentel, P. Melsted & L. Pachter Near-optimal probabilistic RNA-seq quantification. *Nat Biotechnol,* 34 (2016)**,** 525-7.

D. J. Mccarthy, K. R. Campbell, A. T. Lun & Q. F. Wills Scater: pre-processing, quality control, normalization and visualization of single-cell RNA-seq data in R. *Bioinformatics,* 33 (2017)**,** 1179-1186.

M. Pickering, B. W. Pickering, K. J. Murphy & J. J. O'connor Discrimination of cell types in mixed cortical culture using calcium imaging: a comparison to immunocytochemical labeling. *J Neurosci Methods,* 173 (2008)**,** 27-33.

F. Cornelissen, P. Verstraelen, T. Verbeke, I. Pintelon, J. P. Timmermans, R. Nuydens & T. Meert Quantitation of chronic and acute treatment effects on neuronal network activity using image and signal analysis: toward a high-content assay. *J Biomol Screen,* 18 (2013)**,** 807-19.
