# Supplementary material for: Genetically Engineered iPSC-Derived FTDP-17 MAPT Neurons Display Mutation-Specific Neurodegenerative and Neurodevelopmental Phenotypes
Source: Stem Cell Reports. 2018 Jul 26;11(2):363–79. doi: 10.1016/j.stemcr.2018.06.022 (PMC6093179; doi:10.1016/j.stemcr.2018.06.022)
Supplement: Document S2. Article plus Supplemental Information [file mmc2.pdf]

Stem Cell Reports
Article

OPEN ACCESS

Genetically Engineered iPSC-Derived FTDP-17 *MAPT* Neurons Display Mutation-Specific Neurodegenerative and Neurodevelopmental Phenotypes

An Verheyen,[1,*] Annick Diels,[1] Joke Reumers,[1] Kirsten Van Hoorde,[2] Ilse Van den Wyngaert,[1] Constantin van Outryve d'Ydewalle,[1] An De Bondt,[1] Jacobine Kuijlaars,[3] Louis De Muynck,[1] Ronald De Hoogt,[1] Alexis Bretteville,[1] Steffen Jaensch,[1] Arjan Buist,[1] Alfredo Cabrera-Socorro,[1] Selina Wray,[4] Andreas Ebneth,[1] Peter Roevens,[1] Ines Royaux,[1] and Pieter J. Peeters[1]
[1]Janssen Research & Development, A Division of Janssen Pharmaceutica N.V, Turnhoutseweg 30, Beerse 2340, Belgium
[2]Open Analytics NV, Antwerpen 2600, Belgium
[3]Hasselt University, Biomedical Research Institute, Diepenbeek 3590, Belgium
[4]Department of Molecular Neuroscience, Institute of Neurology, University College London, London WC1N 1PJ, UK
*Correspondence: averhey1@its.jnj.com
https://doi.org/10.1016/j.stemcr.2018.06.022

SUMMARY

Tauopathies such as frontotemporal dementia (FTD) remain incurable to date, partially due to the lack of translational *in vitro* disease models. The *MAPT* gene, encoding the microtubule-associated protein tau, has been shown to play an important role in FTD pathogenesis. Therefore, we used zinc finger nucleases to introduce two *MAPT* mutations into healthy donor induced pluripotent stem cells (iPSCs). The IVS10+16 mutation increases the expression of 4R tau, while the P301S mutation is pro-aggregant. Whole-transcriptome analysis of *MAPT* IVS10+16 neurons reveals neuronal subtype differences, reduced neural progenitor proliferation potential, and aberrant WNT/SHH signaling. Notably, these neurodevelopmental phenotypes could be recapitulated in neurons from patients carrying the *MAPT* IVS10+16 mutation. Moreover, the additional pro-aggregant P301S mutation revealed additional phenotypes, such as an increased calcium burst frequency, reduced lysosomal acidity, tau oligomerization, and neurodegeneration. This series of iPSCs could serve as a platform to unravel a potential link between pathogenic 4R tau and FTD.

INTRODUCTION

Tauopathies including frontotemporal dementia (FTD) and Alzheimer disease (AD), are a group of neurodegenerative diseases characterized by the hyperphosphorylation and accumulation of the microtubule-associated protein tau in the human brain (Spillantini and Goedert, 2013). Tau is predominantly expressed in neuronal axons where it controls the polymerization and stabilization of the microtubules while it also regulates axonal transport (Drechsel et al., 1992; Kanaan et al., 2011). However, under pathological conditions, several *MAPT* gene mutations have been linked to hyperphosphorylation and aggregation of tau into neurofibrillary tangles (NFTs) resulting in FTD (Hutton et al., 1998).

Alternative splicing of exon 2, 3, and 10 of the *MAPT* gene on chromosome 17 leads to the expression of six different tau isoforms in the adult human brain, with the longest isoform (2N4R) harboring two N-terminal insertions (exon 2 and 3) and the inclusion of a fourth repeat (exon 10) in tau's microtubule binding domain. The expression of the different isoforms is transcriptionally regulated with only the shortest isoform (0N3R) being expressed at embryonic stages and during development (Kosik et al., 1989). The function and neuronal expression pattern of these different tau isoforms remain to be elucidated, although correct splicing seems to be necessary to keep neurons functional, as unbalanced 4R:3R tau ratios are linked with neurodegenerative disorders such as FTD and Huntington disease (Hutton et al., 1998; Fernandez-Nogales et al., 2014, 2016). For example, *MAPT* mutations that promote the inclusion of exon 10 appear to be sufficient to trigger disease (Hutton et al., 1998).

Over the past years, human induced pluripotent stem cells (iPSCs) have been extensively used to generate better models for human diseases, including neurodegenerative disorders leading to dementia. Human iPSCs can be differentiated into various neuronal subtypes, including cortical neurons, which are known to be affected in FTD (Brun et al., 1994). However, we and others have shown that wild-type iPSC-derived cortical neurons mainly express the embryonic tau isoform that lacks exon 10, even after extended culturing time (Sposito et al., 2015; Verheyen et al., 2015), making it challenging to study exon 10-related tauopathies in this model. Therefore, we introduced the pathogenic IVS10+16 mutation, shown to increase the inclusion of exon 10 (Grover et al., 1999; Sposito et al., 2015) in iPSCs from a healthy donor using zinc finger nuclease (ZFN) technology. Extensive characterization of differentiated IVS10+16 tau neurons compared with the isogenic control revealed neuronal subtype differences accompanied by a reduced neural progenitor (NPC) proliferation

This is an open access article under the CC BY license (http://creativecommons.org/licenses/by/4.0/).

potential and aberrant WNT/SHH signaling, already at the earliest stages of neurodevelopment. Additionally, and with the aim to model an early-onset and more aggressive FTDP-17-linked tauopathy (Bugiani et al., 1999; Baba et al., 2007), we introduced the pro-aggregant P301S point mutation in exon 10. Specific P301S-related phenotypes such as increased calcium burst frequency, reduced lysosomal acidy, tau oligomerization, and neurodegeneration could be observed within weeks after plating of NPCs.

Both *MAPT* IVS10+16 and IVS10+16/P301S iPSCs, in conjunction with their parental isogenic wild-type cells, are useful tools to further elucidate the role of the IVS10+16 mutation, the P301S mutation, and the mechanisms underlying FTDP-17 and other tauopathies.

RESULTS

ZFN-Engineered *MAPT* IVS10+16 and IVS10+16/P301S iPSCs Are Pluripotent and Have a Normal Karyotype

With the aim to generate improved translational human tauopathy models, we used ZFN technology to introduce the FTDP-17-associated *MAPT* IVS10+16 and P301S mutations into commercially available control hiPSCs (iPSC0028) from Sigma. The intronic *MAPT* IVS10+16 mutation was chosen to ensure the inclusion of exon 10 to accelerate and increase the expression of more mature 4R isoforms of tau. The P301S mutation, in *MAPT* exon 10, was selected for its potency to induce tau pathology (Baba et al., 2007; Bugiani et al., 1999). Two single-mutation iPSC lines were generated: mono-allelic IVS10+16 (+/−) and biallelic IVS10+16 (+/+). Next to these single-mutant lines, also a double-mutant IVS10+16 (+/+)/P301S (+/+) iPSC line, biallelic for both mutations, was generated to ensure the expression of P301S in all 4R tau isoforms. The genetically engineered iPSCs were fully characterized. G-banding reveals a normal karyotype (Figure 1A), while pluripotency is confirmed using immunofluorescent staining for OCT4 and NANOG (Figure 1B). Finally, Sanger sequencing confirms the presence of the mono- and biallelic IVS10+16 mutation and the biallelic P301S point mutation (Figure 1C).

IVS10+16 Tau Neurons Display Increased Inclusion of Exon 10 on mRNA and Protein Levels Compared with the Isogenic Control

To validate whether the intronic *MAPT* IVS10+16 mutation increases the inclusion of exon 10, reflected by increased 4R tau expression, neurons were generated using a well-established cortical differentiation protocol (Kuijlaars et al., 2016; Shi et al., 2012). The inclusion of *MAPT* exon 10 and the amount of total tau were evaluated over time at the mRNA level using RT-qPCR. As previously shown by us and others (Sposito et al., 2015; Verheyen et al., 2015), we failed to detect 4R tau mRNA (*MAPT* exon 10) in control neurons at day *in vitro* (DIV) 80. In contrast, in all *MAPT* IVS10+16 and IVS10+16/P301S clones, 4R tau mRNA is expressed from the NPC stage onward, with increasing levels after further differentiation into neurons (Figure 2A). The mRNA for total *MAPT* and 3R tau (*MAPT* without exon 10) also increases over time (Figures 2B and 2C). The presence of tau protein is confirmed using western blot and immunofluorescent staining (Figures 2D and 2E), and shows no difference between control and mutant neurons at DIV80. However, there is more tau protein expressed in IVS10+16 mutant neurons at earlier time points (DIV51 and DIV65; $p < 0.0001$ for both; Figure S1) and in general there is more tau protein expressed in neurons compared with NPCs ($p < 0.0001$ for both control and IVS10+16 neurons; Figure S1). When looking at tau phosphorylation, we observe increased levels of phosphorylation at epitopes Ser396/Ser404 (PHF1) and Thr181 (AT270) in IVS10+16 neurons compared with control neurons 5 and 7 weeks after plating (DIV65 and DIV80; $p < 0.05$, IVS10+16 versus control; Figure S1). Finally, neuronal expression of 4R tau protein is confirmed by western blot with a 4R tau-specific antibody and with a total tau antibody in combination with λ phosphatase treatment (Figures 2F and 2G).

Whole-Transcriptome Analysis Reveals Differences in Neuronal Subtypes and Cell-Cycle-Related Transcription Factors in IVS10+16 Neurons

To explore the potential effect of *MAPT* IVS10+16 and P301S mutations separately, all mutant and isogenic control iPSC-derived NPCs and neurons were subjected to microarray analysis. Principal component analyses of DIV31 NPCs and DIV65 neurons reveal that the largest proportion of all differentially expressed genes is linked to the IVS10+16 mutation (47% principal component 1 [PC1] at DIV31 and 78% PC1 at DIV65), while a smaller percentage corresponds to differences due to the P301S mutation (spectral maps in Figures 3A and 3B). Therefore, we first focused on the differences between control and mono-allelic IVS10+16 (+/−) neurons and found that 10.98% of all tested genes between these two groups are significantly different at the NPC stage, while 34.47% are significantly different at DIV65 (Volcano plots; Figures 3A and 3B). Gene Ontology Biological Processes (GOBP) such as forebrain cortex development, limbic system and hippocampus neuronal development, forebrain regionalization, and neuron fate commitment are altered, suggesting that control and mutant neurons correspond to different neuronal cell types that may be representative for different brain areas. This is reflected by a significantly reduced expression of forebrain and glutamatergic markers in IVS10+16 neurons (Figures 3C and S2 and Table S1). On

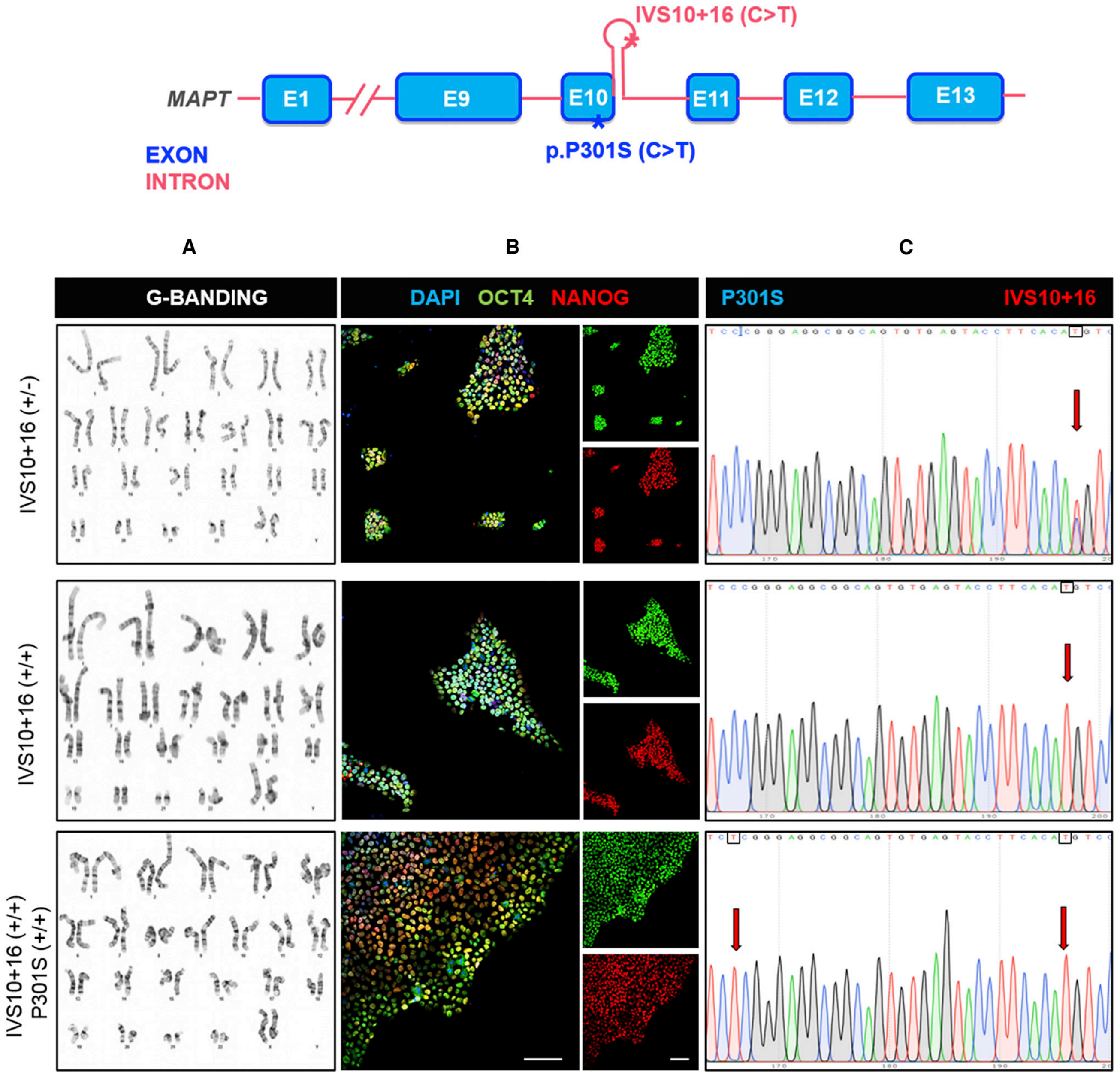

Figure 1. ZFN-Engineered *MAPT* IVS10+16 and IVS10+16/P301S iPSCs Are Pluripotent and Have a Normal Karyotype
(A) G-banding shows a normal karyotype for all three mutant iPSC lines.
(B) Immunofluorescent staining for the pluripotency markers OCT4 and NANOG in combination with the nuclear marker DAPI. Scale bars represent 100 μm.
(C) The result of Sanger sequencing showing the iPSC clones with mono-or biallelic IVS10+16 (C>T) mutations and the iPSC clone with a biallelic P301S (C>T) mutation and biallelic IVS10+16 mutation. (C>T) mutations are indicated with red arrows.

the other hand, limbic, GABAergic, and basal ganglia markers are upregulated in all mutant IVS10+16 neurons (Figures 3D and S2 and Table S1). Immunofluorescent staining and quantification of TBR1, VGlut2, ISL1, and NKX2-1 proteins confirms the difference in neuronal subtypes between control and IVS10+16 neurons (Figure 3E).

Furthermore, top affected Gene Ontology Molecular Functions (GOMF) at DIV31 and DIV65 include RNA polymerase II activating transcription factor, E-box binding, and transcriptional repressor activity. Several transcription factors and cell-cycle-related genes are significantly different in IVS10+16 neurons (Figure 4A and Table S1),

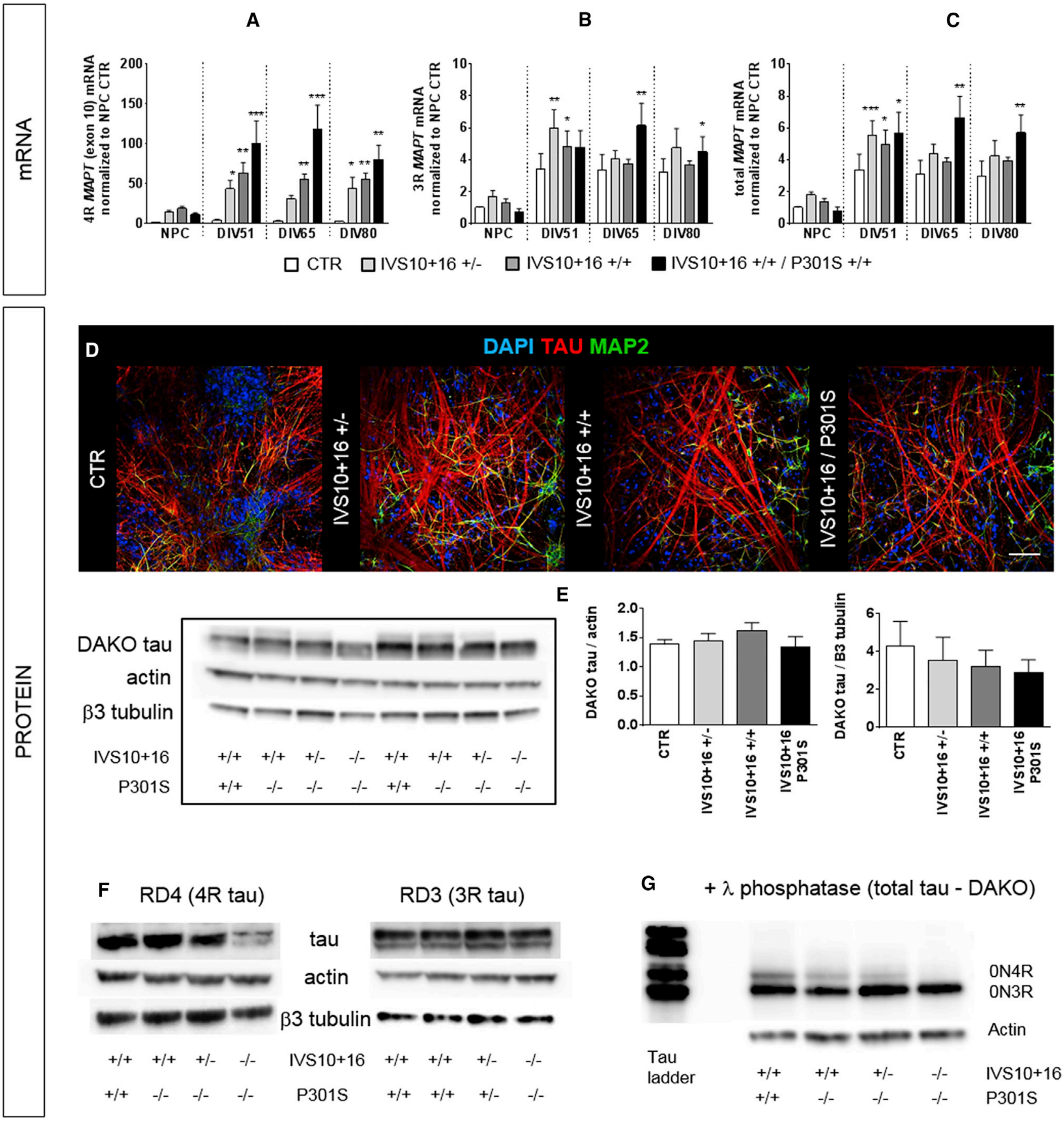

Figure 2. *MAPT* IVS10+16 Neurons Display Inclusion of Exon 10 at mRNA and Protein Level

(A–C) RT-qPCR on NPCs (DIV31) and differentiated neurons at different time points, using primers detecting: 4R tau, *MAPT* exon 10 (A); 3R tau, *MAPT* exon 9–11 (B); or total tau, total *MAPT* (C). Neurons were lysed 3, 5, and 7 weeks after final plating of NPCs and correspond to DIV51, DIV65, and DIV80 from iPSC differentiation. $^{*}p < 0.05$, $^{**}p < 0.01$, and $^{***}p < 0.001$ (compared with NPC control, Kruskal-Wallis test with Dunn's post hoc analysis, n = 3). CTR, control.

(D) Immunofluorescent staining with antibodies against MAP2 and TAU (DAKO) on mutant and control (DIV51) neurons. DAPI was used to stain the nucleus. Scale bar represents 100 μm.

(E) Western blot for total tau (DAKO) shows no difference between control and mutant neurons (DIV80; n = 4; p = non-significant [NS], 1-way ANOVA).

(legend continued on next page)

suggesting a potential difference in the proliferation potential of IVS10+16 NPCs. To test the proliferation potential of mutant and control NPCs, 5-ethynyl-2′-deoxyuridine (EdU) staining was performed in combination with the nuclear marker DAPI and the neuronal marker β3-tubulin. Neurons with the IVS10+16 mutation (DIV51) display a significantly reduced percentage of EdU-positive cells ($p < 0.01$; $n = 3$), less cells in general (DAPI counts; $p < 0.01$; $n = 3$), and an increased β3-tubulin-positive area compared with control neurons, while the same number of NPCs was plated (Figures 4B and 4C).

Finally, the WNT protein binding pathway (GO:0017147) is also affected in IVS10+16 mutant NPCs and neurons. At the NPC stage, there are decreased transcript levels of *WNT*-related genes such as *WLS*, *DKK3*, *WNT7B*, *FZD7*, *ROR1*, and *APCDD1*, while *WNT7A* and *FZD5* transcripts are upregulated (Table S2 and Figure S2). At DIV65, there is upregulation of some additional WNT-related genes such as *GSK3B*, *WNT4*, *AXIN2*, *FZD3*, while *FZD8* and *FZD2* are downregulated. Notably, in IVS10+16 neurons, the WNT ligand secretion mediator *WLS* is downregulated at the NPC stage and upregulated at DIV65, while *SFRP1* is upregulated in NPCs and downregulated in differentiated neurons. When looking at differentially expressed genes of the sonic hedgehog (SHH) pathway, we found that *LRP2*, *PTCH1*, *PTCHD1* are significantly upregulated in mutant NPCs and neurons, while *CEP76* and *CAV1* are downregulated (Table S2 and Figure S2). These data suggest that WNT and SHH signaling pathways are different in control and IVS10+16-derived neural cells and that there is a complex regulation of the genes that are involved in these pathways.

To evaluate whether these observed phenotypes are a consequence of 4R tau expression, we used adeno-associated viruses (AAV6) to overexpress the longest isoform of 4R tau (2N4R) or a negative control (GFP) in control NPCs followed by evaluation of proliferation potential and some selected genes involved in cell cycle control and WNT/SHH pathways. Since these NPCs (DIV31) were already "primed" toward a cortical fate, we did not evaluate neuronal subtype changes after 4R tau overexpression. Three weeks after transduction of control cortical NPCs, there is no difference in proliferation potential and mRNA levels of the selected genes between 4R tau overexpressing neurons and controls (Figure S3). This suggests that, in our system, the observed phenotypes are not due to the expression of 4R tau.

Heterogeneous Expression of *MAPT* with and without Exon 10 in Human Cortex at the Single-Cell Level

The *MAPT* IVS10+16-induced neuronal subtype changes in our *in vitro* neuronal model prompted us to look at tau isoforms with and without exon 10 in the neuronal population of a healthy human cortex. Therefore, we used a publicly available dataset generated recently by Lake et al. (2016). For their study, single-cell transcriptome analysis was performed on isolated nuclei from postmortem human cerebral cortex. Unbiased principal component analysis separates interneurons and excitatory neurons into two major clusters, after color mapping with the glutamatergic marker SLC17A7 and the GABAergic marker GAD1 (Figure 5A). When coloring the summated isoforms of *MAPT* (*ENSEMBL* ENSG00000186868) with exon 10 (*MAPT_ex10*) and *MAPT* without exon 10 (*MAPT_no_ex10*), there was no clear separation into cell clusters and we did not find a correlation of *MAPT_ex10* or *MAPT_no_ex10* with any of the glutamatergic or interneuron markers tested (Figures 5B and S4). However, we observed a positive correlation at the single-cell level between the interneuron markers *GAD1* and *GAD2* as well as between the excitatory glutamatergic genes *SLC17A7*, *TBR1*, and *SATB* and a negative correlation between the interneuron and excitatory markers, suggesting good quality of the data and the analysis tools used (Figure 5B). This suggests that, in the adult human cortex, 4R tau mRNA is present in both interneurons and excitatory neurons, although with different expression levels.

Neurons of Patients with the IVS10+16 Mutation Display a Similar Phenotype as ZFN-Gene-Edited IVS10+16 Neurons

As we were unable to link our observed phenotypes to 4R tau expression only, we evaluated whether these phenotypes could be confirmed in FTDP-17 patient-derived cells with the same IVS10+16 mutation. Therefore, iPSCs from two different *MAPT* IVS10+16 patients (Sposito et al., 2015) were differentiated in parallel with our ZFN-gene-edited *MAPT* IVS10+16 (+/−) and control iPSCs and subjected to microarray analysis. Only the mono-allelic ZFN-gene-edited iPSC line was chosen since the mutation is mono-allelic in patients as well. The result of this analysis shows that a large part of the variance (PC1 = 56%) is explained by the presence or absence of exon 10 (Figure 6A) as all three mutants cluster together at the opposite site of the Sigma control. The remainder (PC2 = 23%) of the variance is covered by differences among the two different patients

(F) Western blot on DIV65 control and mutant neurons using specific 3R tau and 4R tau antibodies. For (E) and (F), actin and β3-tubulin antibodies were used as loading control.
(G) Western blot on DIV65 control and mutant neurons using the DAKO total tau antibody shows presence of both 3R and 4R tau isoforms in mutant neurons, after λ phosphatase treatment. A tau ladder with all six isoforms is included.
See also Figure S1.

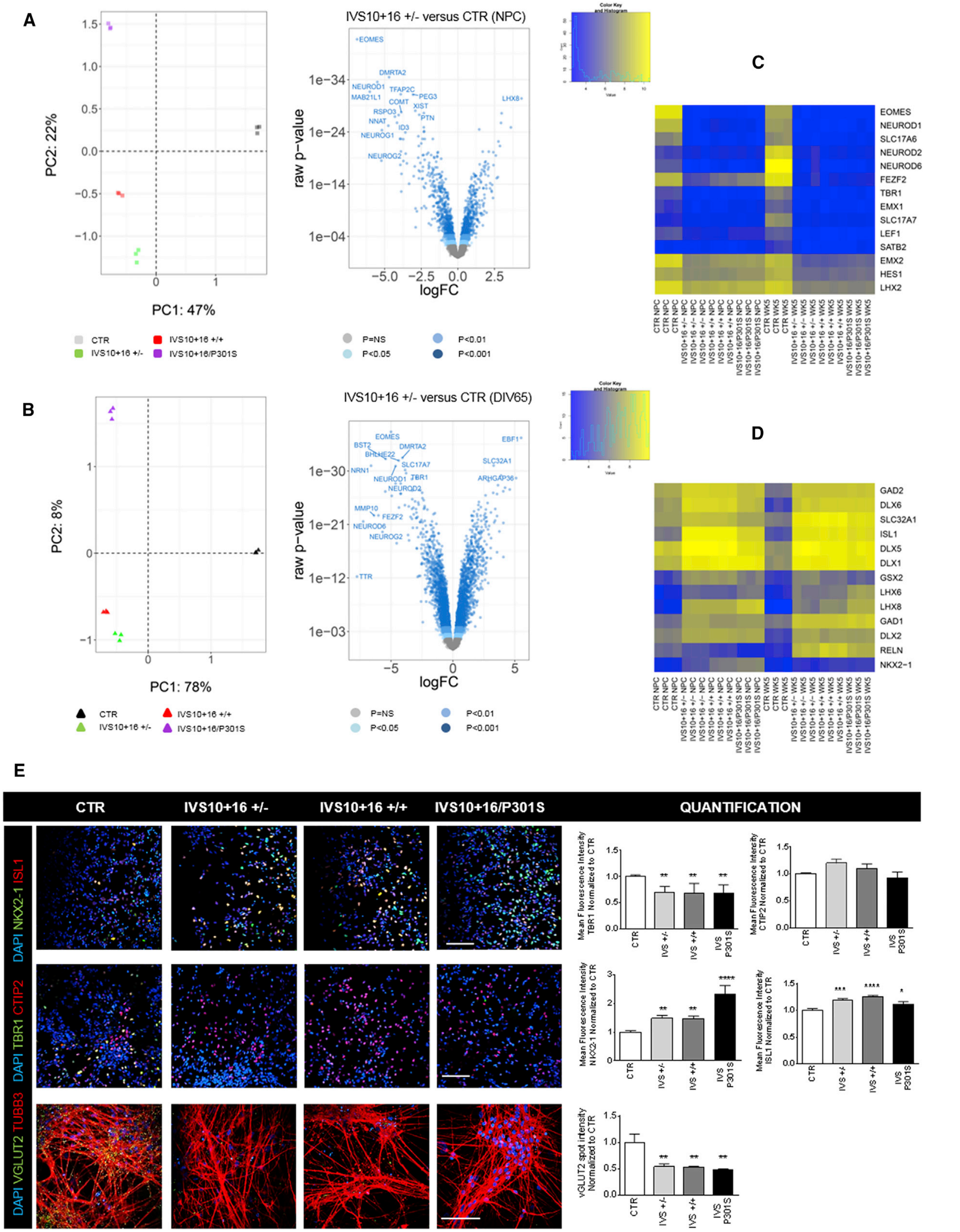

(legend on next page)

and the ZFN-edited neurons. Overlap analysis between all three mutant neurons (DIV65; patient and ZFN edited) compared with the Sigma parental control, revealed that 1,983 genes are upregulated and 2,518 genes are downregulated in all mutants compared with the control (Figure 6B). Notably, the previously identified GOBP and GOMF pathways that are affected in the gene-edited lines are confirmed in these patient-derived neurons. Likewise, these patient-derived neurons show a reduction in glutamatergic genes and an upregulation of GABAergic markers together with differentially expressed transcription factor genes, WNT/SHH signaling, and WNT protein binding pathways (Figure 6C and Table S2) compared with the Sigma control.

Finally, we also evaluated whether some other well-known neurodegenerative disease-related genes are affected in our *MAPT* IVS10+16 mutant NPCs and neurons, compared with the control. The AD-related *APOE* gene is significantly downregulated in all mutant lines. When looking at *MAPT* exon 10 splicing mediators (reviewed in Park et al., 2016), we detect differential expression of *SRSF1*, *SRSF3*, *SRSF4*, *SRSF6*, *SRSF7*, *CELF3*, *CELF4*, *NOVA1*, and *SWAP70* (Table S3), confirming that the splicing machinery around *MAPT* exon 10 is differentially regulated in IVS10+16 mutant and control cells. Regarding FTD-related genes, we found a significant increase in *TARDBP* (TDP-43) at DIV65, while *GRN* was significantly lower at the NPC stage in all *MAPT* IVS10+16 cells (Table S4). Remarkably, the Parkinson disease (PD)-related gene *CHCHD2* is also significantly downregulated in all mutant lines, while *GAK* is upregulated.

Thus, both ZFN-engineered and patient-derived IVS10+16 neurons show overlapping expression patterns with regard to neuronal subtype definition; WNT/SHH signaling; transcription factor expression; and some selected AD, PD, and FTD-related genes.

Increased Burst Frequency in IVS10+16 Neurons with the Additional P301S Mutation

Aiming for a more pronounced FTDP-17-related phenotype, we generated a double-mutant iPSC line, adding the pro-aggregant P301S mutation in *MAPT* exon 10 on top of the intronic IVS10+16 mutation. Since the mutation was introduced on a biallelic IVS10+16 background, only the IVS10+16 biallelic single-mutant line, expressing both wild-type 3R and 4R tau and largely consisting of the same neuronal subtypes (Figure 3), was chosen as a control for comparisons in this set of experiments. Microarray data reveal that 1,341 genes are differentially expressed at the NPC stage (DIV31), 664 genes at DIV51, 574 genes at DIV65, and 762 genes at DIV80 (Figure S5). The most prominently affected function at the NPC stage (GOMF) reveals calcium channel regulator activity, represented by downregulation of CACNA2D3, *NRXN3*, and *PRKCB* at all time points, while *S100A10* is upregulated (Figure S5 and Table S5). To study a potential effect on calcium activity, live cell calcium imaging with Fluo-4 was performed on neurons with and without P301S mutation. The burst frequency of the neurons with P301S mutation is significantly higher ($p < 0.01$, $n = 3$) than in neurons expressing wild-type tau isoforms (Figure 7A), suggesting a hyperexcitable phenotype.

P301S-Carrying Neurons Display Tau Oligomerization after Seeding with K18 Fibrils

Both patient-based and experimental evidence (Baba et al., 2007; Guo and Lee, 2013; Bugiani et al., 1999; Sperfeld et al., 1999) have shown the presence of tau aggregates and NFTs in P301S neurons. In our wild-type and P301S mutant neurons, we did not observe spontaneous formation of tau aggregates after an extended culturing period (DIV100) (not shown), measured by the highly sensitive AlphaLISA technology as previously described (Verheyen et al., 2015; Medda et al., 2016). Therefore, we decided to trigger oligomerization or aggregation by adding recombinantly expressed preformed human mutant (P301L) K18 fibrils, representing the 4R tau microtubule binding domain that has been described to seed tau aggregation (Guo and Lee, 2013; Verheyen et al., 2015). AlphaLISA to detect oligomerization and aggregation of tau (hTAU10/10) already

Figure 3. Neuronal Subtype Differences Related to the *MAPT* IVS10+16 Mutation, by Whole-Transcriptome Analysis

(A and B) Microarray on control and mutant NPCs (A) and DIV65 neurons (B) followed by principal component analysis reveals that the largest difference (47% at NPC stage and 78% at neuron stage; n = 3 biological replicates) is due to the presence of the IVS10+16 mutation while a lower percentage of differences is explained by the P301S mutation (22% at NPC stage and 8% at neuron stage; n = 3 biological replicates). PC, principal component.

(C and D) Heatmaps showing the separate clustering of control and all IVS10+16 mutant cells regarding glutamatergic and GABAergic markers.

(E) Immunofluorescent staining on control and mutant NPCs and neurons shows an increase of NKX2-1 and ISL1 proteins in IVS10+16-carrying NPCs (DIV31) (NKX2-1, $p < 0.0001$, $n = 3$; and ISL1, $p < 0.0001$, $n = 3$) and a reduction of TBR1 and VGlut2 proteins in IVS10+16-carrying neurons (DIV51-65) (TBR1, $p = 0.0038$, $n = 3$; and VGlut2, $p = 0.0026$, $n = 3$), compared with the control (CTR). CTIP2 is present similarly in both control and mutant neurons (p = NS; $n = 3$). Scale bar represents 100 μm. One-way ANOVA for ISL1, TBR1, VGlut2, and CTIP2, and Kruskal-Wallis test for NKX2-1. *$p < 0.05$, **$p < 0.01$, ***$p < 0.001$, ****$p < 0.0001$.

See also Table S1 and Figures S2 and S3.

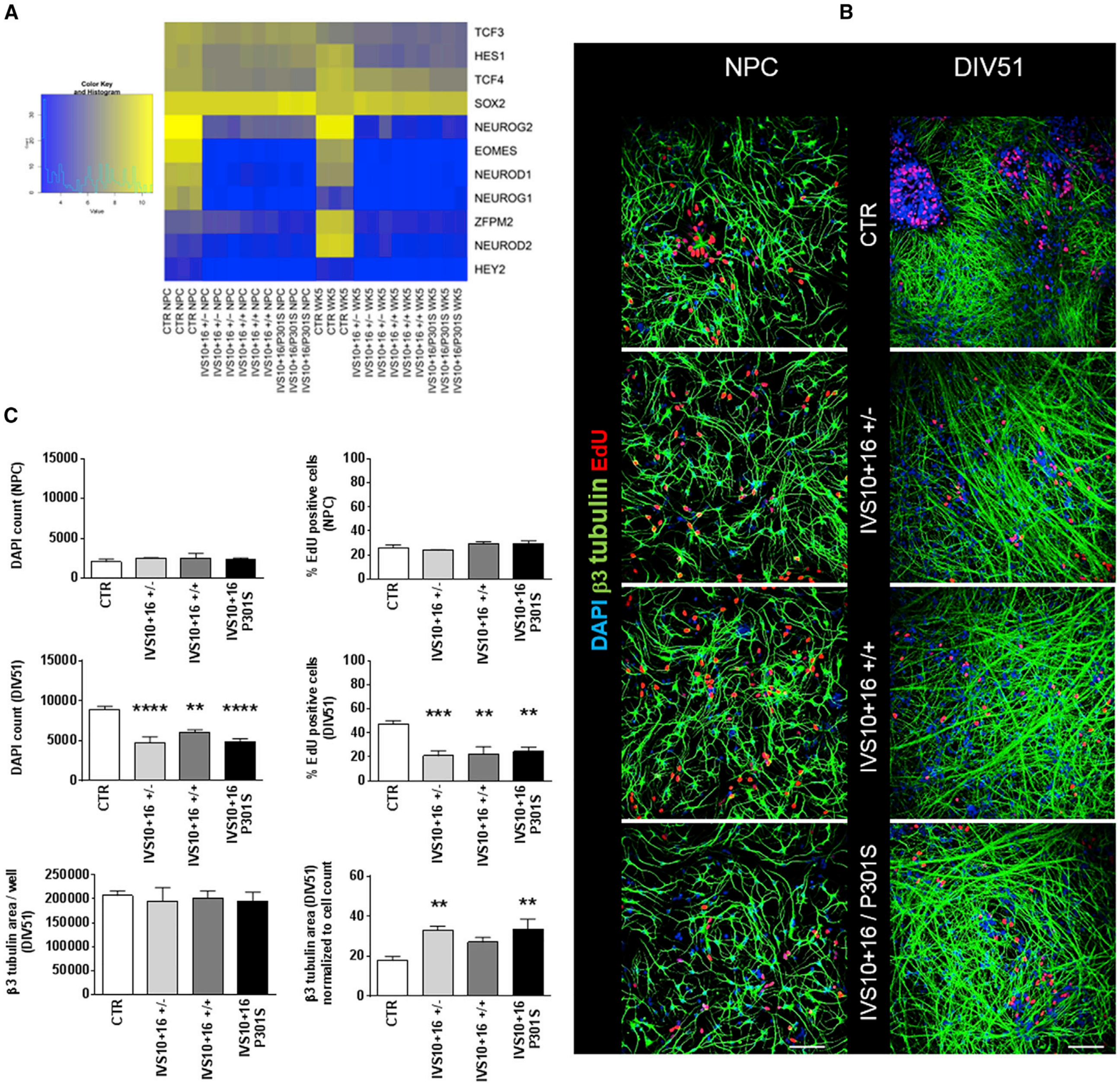

Figure 4. *MAPT* IVS10+16 NPCs Display a Reduced Proliferation Potential

(A) Heatmap showing the differences in clustering between control and IVS10+16 NPCs and neurons regarding transcription factor expression.

(B and C) Immunofluorescent EdU staining in combination with the neuronal marker β3-tubulin and nuclear marker DAPI. There is no difference in the amount of DAPI and EdU-positive cells at the NPC stage (n = 3; p = NS) while there is a significant reduction of proliferating cells in all mutant lines 3 weeks after plating (DIV51; n = 3; $p < 0.001$), and an increase in β3-tubulin-positive area normalized to cell count (DIV51, n = 3; p = 0.0075). Scale bar represents 100 μm. **$p < 0.01$, ***$p < 0.001$, and ****$p < 0.0001$ one-way ANOVA for DIV51 samples and Kruskal-Wallis for NPCs.

See also Table S1 and Figure S3.

shows an increased signal ($p < 0.0001$; n = 3) in neurons expressing P301S tau and treated with K18-P301L seeds 5 weeks after seeding, but not in wild-type IVS10+16 neurons (Figure 7B). Note that there is also no increase in AlphaLISA signal when wild-type neurons are treated with wild-type K18 seeds (Figure S6). With the aim to better

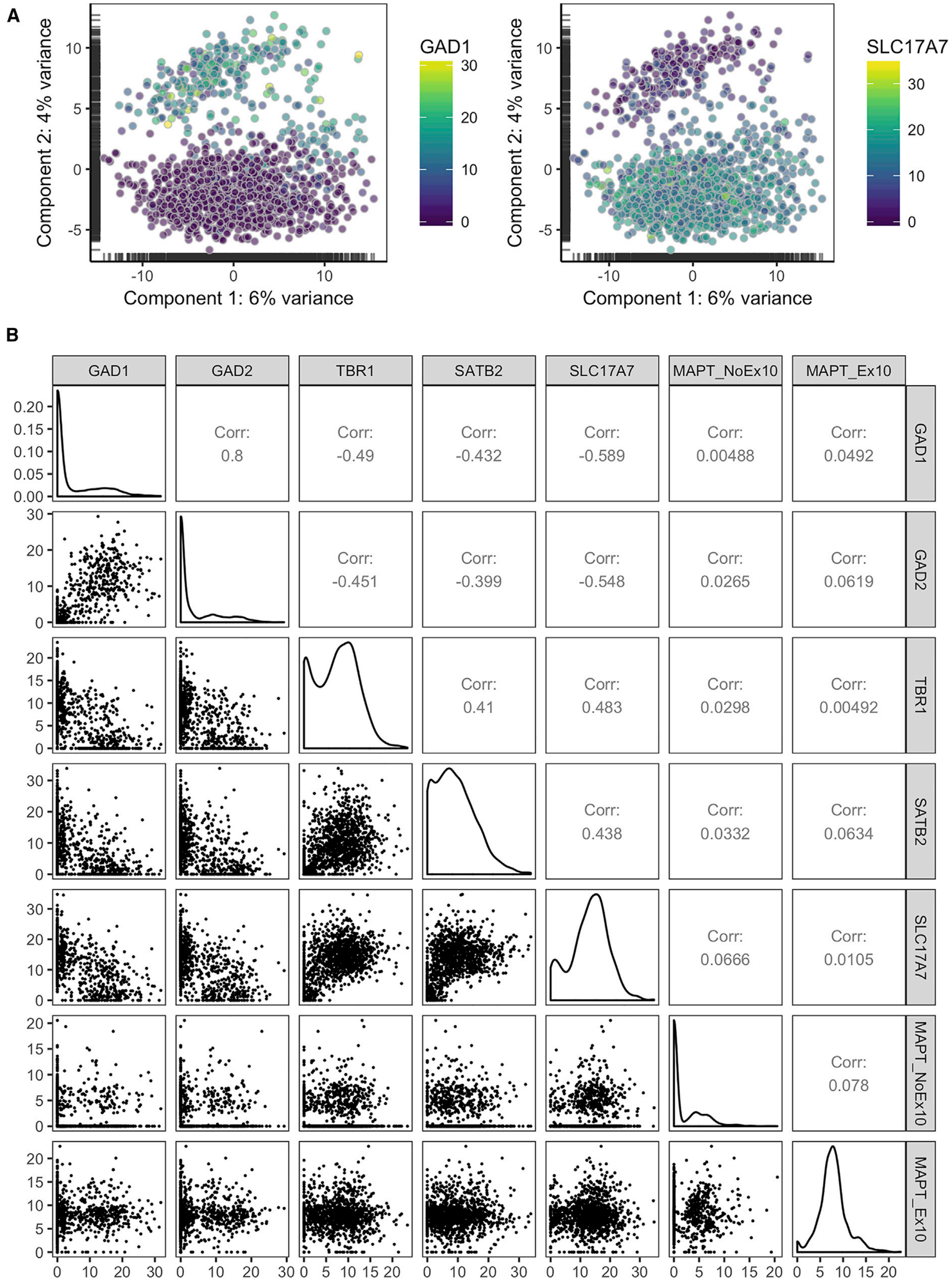

(legend on next page)

characterize the nature of this increased hTAU10/10 signal, Sarkosyl extraction followed by western blot (HT7 antibody) was performed on IVS10+16/P301S neurons with and without K18-P301L fibrils. We failed to detect insoluble tau (Figure S6C), which is in contrast to what we have previously shown in our virally induced tau aggregation model (Verheyen et al., 2015). Therefore, this might suggest that the increased AlphaLISA signal in the current model more likely reflects the presence of dimers or oligomers in P301S-carrying neurons. AlphaLISA for total tau (HT7/hTAU10) shows that total tau levels are not affected by K18 seeding (Figure 7C). The selective increase in the hTAU10/10 AlphaLISA signal in P301S mutant neurons is not due to a difference in uptake of the K18 seeds, as a similar intensity of pHrodo-labeled K18 is detected in both control (IVS10+16) and IVS10+16/P301S neurons (p = non-significant, n = 4) (Figure 7D). However, a decreased LysoTracker spot intensity is observed in DIV65 IVS10+16/P301S mutant neurons compared with IVS10+16 control neurons, indicative of an increased lysosomal pH ($p < 0.0001$; n = 4) (Figure 7E). To evaluate whether the above-described P301S-related increases in burst frequency and lysosomal pH affect the general health of the neurons, the number of apoptotic cells and the neuronal network area were quantified using TUNEL staining and β3-tubulin staining. Indeed, more apoptotic neurons are counted in DIV65 P301S-carrying neurons (Figure 7F), compared with their respective control (p = 0.042; n = 3), without affecting the neuronal network at this time point (Figure 7G).

DISCUSSION

We have successfully engineered iPSC lines containing the *MAPT* IVS10+16 mutation with and without additional P301S mutation using ZFN technology. The pathological IVS10+16 mutation was selected for its potency to fasten the inclusion of *MAPT* exon 10 (Grover et al., 1999), while the pro-aggregant P301S mutation was chosen to generate an aggressive FTDP-17 model to complement several published *in vitro* and *in vivo* tau P301S systems (Guo and Lee, 2013; Malmanche et al., 2017; Yoshiyama et al., 2007).

Differentiated neurons with the *MAPT* IVS10+16 mutation expressed 4R tau at both mRNA and protein levels, in alignment with published data on IVS10+16 patient iPSC-derived neurons (Imamura et al., 2016; Sposito et al., 2015). Moreover, since *MAPT* IVS10+16 neurons express wild-type 4R tau, these neurons provide the best control for our double-mutant *MAPT* IVS10+16/P301S neurons, expressing 4R tau with the P301S mutation, and provide the opportunity to study mutation-specific phenotypes.

The *MAPT* IVS10+16 Mutation Drives Neurodevelopmental Phenotypes

Whole-transcriptome analysis, evaluating the effects of the *MAPT* IVS10+16 mutation, revealed differences in forebrain cortex development, limbic system, and neural fate commitment as the main affected pathways. These data suggest that IVS10+16 mutant and control neurons represent different neuronal subtypes reminiscent of different parts of the brain. The reduction of cortical glutamatergic markers and the increase of interneuron and basal ganglia markers was confirmed at the protein level. These findings could be confirmed in iPSC-derived neurons from two different IVS10+16 patients, suggesting that these effects are not due to an off-target effect of the ZFN technology. An increasing amount of evidence suggests that tau isoforms with and without exon 10 might be differentially expressed in different neuronal subtypes, although with an overall ratio (4R/3R tau) of one in the whole brain. For example, mRNA expression data in different healthy brain regions revealed that the globus pallidus has the highest relative 4R tau expression, while the frontal cortex shows the lowest 4R tau expression (Majounie et al., 2013). Furthermore, 4R tau expression in iPSC-derived cortical neurons seems to take a long time (Iovino et al., 2015; Sposito et al., 2015), while iPSC-derived dopaminergic neurons express different 4R tau isoforms within a relatively short time frame (Beevers et al., 2017), suggesting that different neuronal subtypes express different levels of the different tau isoforms at certain time points. In this study, we analyzed data from single-cell RNA sequencing on healthy adult postmortem cerebral cortex generated by Lake et al. (2016) and found a huge variation in the amount of *MAPT* transcripts with and without exon 10, independently of the neuronal subtype. Both excitatory neurons and interneurons expressed 4R tau at variable levels. Note that, for our analyses, we did not consider potential differences

Figure 5. Single-Cell RNA Sequencing Analysis of Cerebral Cortex Shows Heterogeneous Expression of *MAPT* with and without Exon 10

(A) Principal component analysis shows clustering of single cells into two populations that are positive for either the interneuron marker *GAD1* or the excitatory marker *SLC17A7*, when highlighting these markers.

(B) Correlation plots showing GABAergic markers *GAD1* and *GAD2*; the excitatory genes *SLC17A7*, *TBR1*, and *SATB2*; and summated *MAPT* isoforms either with or without exon 10. There is no correlation between any of the neuronal subtype markers and *MAPT* isoforms.

See also Figure S4. Corr., correlation.

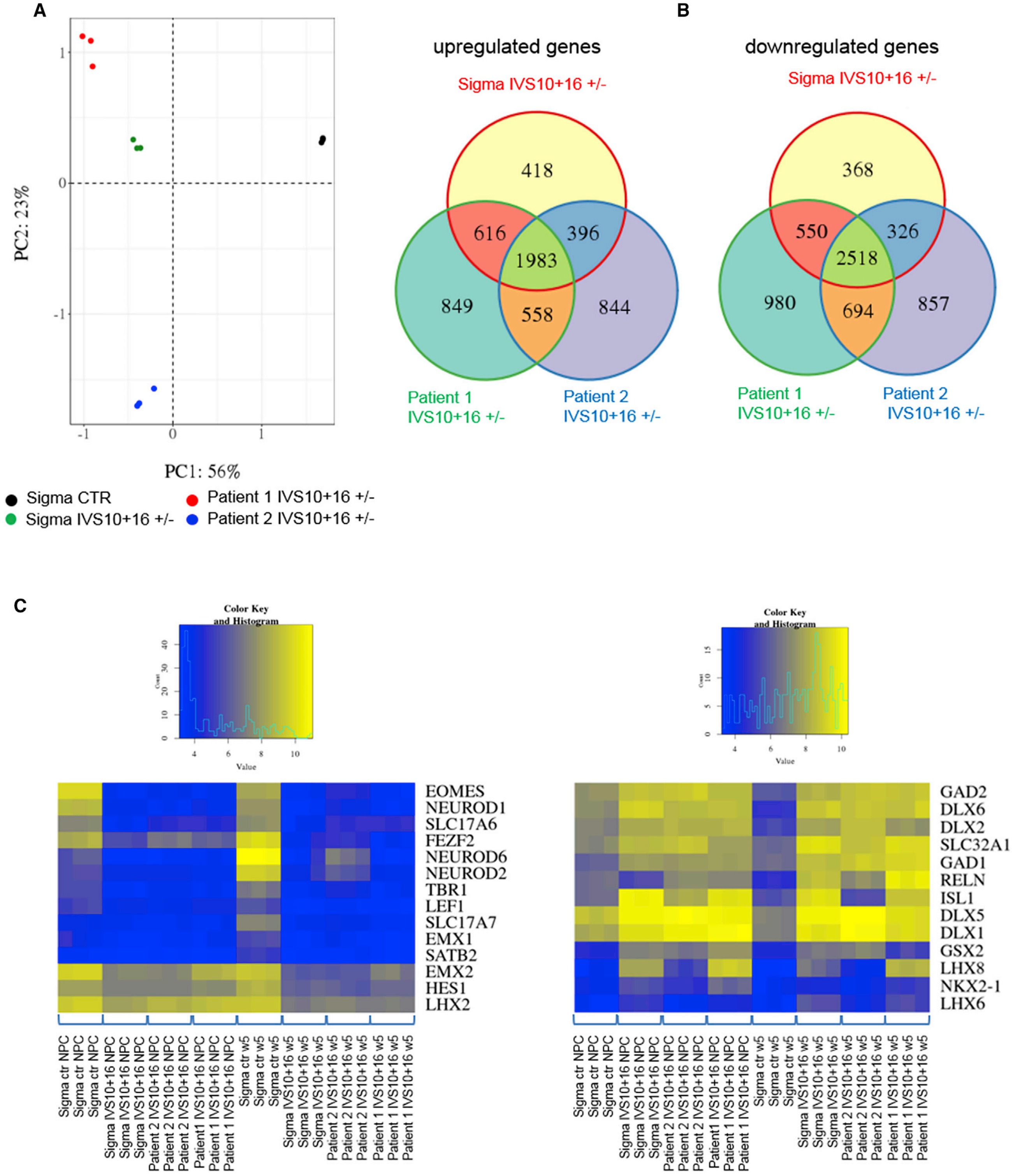

Figure 6. Patient-Derived *MAPT* IVS10+16 Neurons Display a Similar Phenotype as ZFN-Gene-Edited *MAPT* IVS10+16 Neurons
(A) Microarray on control, ZFN-gene-edited, and two different patient-derived *MAPT* IVS10+16 neurons (DIV65) followed by principal component analysis (PC1 = 56% and PC2 = 23%; n = 3 biological replicates).

(legend continued on next page)

between Brodmann areas. More in-depth single-cell transcriptomics analysis on all different human brain areas and at different stages of brain development would provide more insight into the expression pattern of all tau isoforms in adult brain and during brain development.

Besides neuronal subtype differences, a reduced proliferation of IVS10+16 NPCs is observed and an increased β3-tubulin-positive neuronal network area, suggesting faster differentiation and maturation of IVS10+16 neurons, which is in line with the early maturation of mutant *MAPT* iPSC neurons that has been observed before, although with different mutations (Iovino et al., 2015). In our study, the reduced proliferation capability could not be linked to the expression of 4R tau as AAV6-induced overexpression of 4R tau did not reduce the proliferation potential compared with controls. This is in contrast to what has been published before in some *in vitro* and *in vivo* studies (Sennvik et al., 2007; Chen et al., 2010) and is potentially due to differences in the various systems used.

An increasing amount of evidence suggests that neurodegenerative diseases such as AD and FTD are linked with aberrant WNT signaling (Riise et al., 2015; Rosen et al., 2011; Korade and Mirnics, 2011). Also in our study, the WNT signaling pathway is affected in neurons (and NPCs) with the IVS10+16 mutation. Several *WNT*, *SHH*, and *FZD* genes are differentially expressed and *GSK3β* is significantly upregulated in IVS10+16 tau-expressing neurons, reinforcing the link between FTDP-17 mutations, tau pathology, and GSK3β. This differential expression takes place as early as in the developmental stage, long before the formation of NFTs.

The Additional P301S Mutation in 4R Tau Induces Additional Phenotypes that Can Be Associated with Neurodegeneration

We also characterized the ZFN-engineered double-mutant IVS10+16/P301S neurons in comparison with the single-mutant IVS10+16 neurons. Live cell calcium imaging revealed an increased burst frequency, suggesting hyperexcitability of the neurons carrying the P301S mutation. These data are in line with published studies showing neuronal hyperexcitability and epileptic seizures in P301S animal models and FTDP-17 patients with this mutation (Garcia-Cabrero et al., 2013; Sperfeld et al., 1999).

Another well-recognized characteristic of P301S-related FTDP-17 is tau hyperphosphorylation and the formation of NFTs. Although we did not see spontaneous formation of tau aggregates in our neuronal cultures (up to DIV100), we could induce tau oligomerization after seeding with K18 (P301L) in 4R tau-expressing neurons with the P301S mutation. Notably, the AlphaLISA hTAU10/10 signal was remarkably lower than in the AAV-induced iPSC-neuronal model that we have previously reported (Verheyen et al., 2015) and we did not find evidence for the presence of insoluble tau, suggesting that soluble dimers or smaller oligomers are present in our current model. More in-depth analysis using more specialized technologies would be helpful to further characterize these non-monomeric tau species. Potentially, this lower hTAU10/10 AlphaLISA signal is due to the relatively low levels of 4R tau protein compared with 3R tau protein in our neurons at the chosen time points. Alternatively, the difference in neuronal subtypes might play a role as well, as excitatory neuronal cell types might intrinsically be more prone to the formation of NFTs.

Furthermore, the increased lysosomal pH that we observed in P301S-expressing neurons potentially affects the clearance of tau oligomers and formation of NFTs at a later stage, as suggested by others using tau-expressing cell lines (Guo et al., 2016; Xu et al., 2016). Finally, increased apoptosis was observed in P301S-carrying neurons, potentially resulting from the hyperexcitability and increased lysosomal pH and in line with published findings (Lopez-Gonzalez et al., 2015; Yoshiyama et al., 2007).

Summary and Conclusion

FTDP-17 linked with tau is a complex neurodegenerative disorder, with a mutation- and patient-dependent plethora of neurological and clinical manifestations (reviewed in Ghetti et al., 2015) and with different areas of the brain being affected. In the present study, we observed neuronal subtype differences due to the IVS10+16 mutation, which might highlight the neuronal subtypes and cortical layers that are predominantly affected in these patients, such as the medial temporal lobe (limbic system) and basal ganglia (Whitwell et al., 2009).

Since *MAPT* IVS10+16 FTD patients inherently carry the mutation already during developmental stages, there must be compensatory mechanisms such as morphogens or growth factors by supporting cell types in the brain that keep patients cognitively normal until the onset of disease. However, a neurodevelopmental predisposition to dementia has been observed in non-demented pre-symptomatic family members with an FTDP-17 mutation (Geschwind et al., 2001), and microdeletions surrounding the *MAPT* locus have been linked to intellectual disability, suggesting that tau might be involved in the regulation of early

(B) Overlap analysis between all mutants compared with control. In all three mutants, 1,983 common genes are upregulated and 2,518 common genes are downregulated compared with the control.
(C) Heatmaps showing the separate clustering of control and mutant cells, focusing on glutamatergic and GABAergic markers.
See also Tables S2–S4.

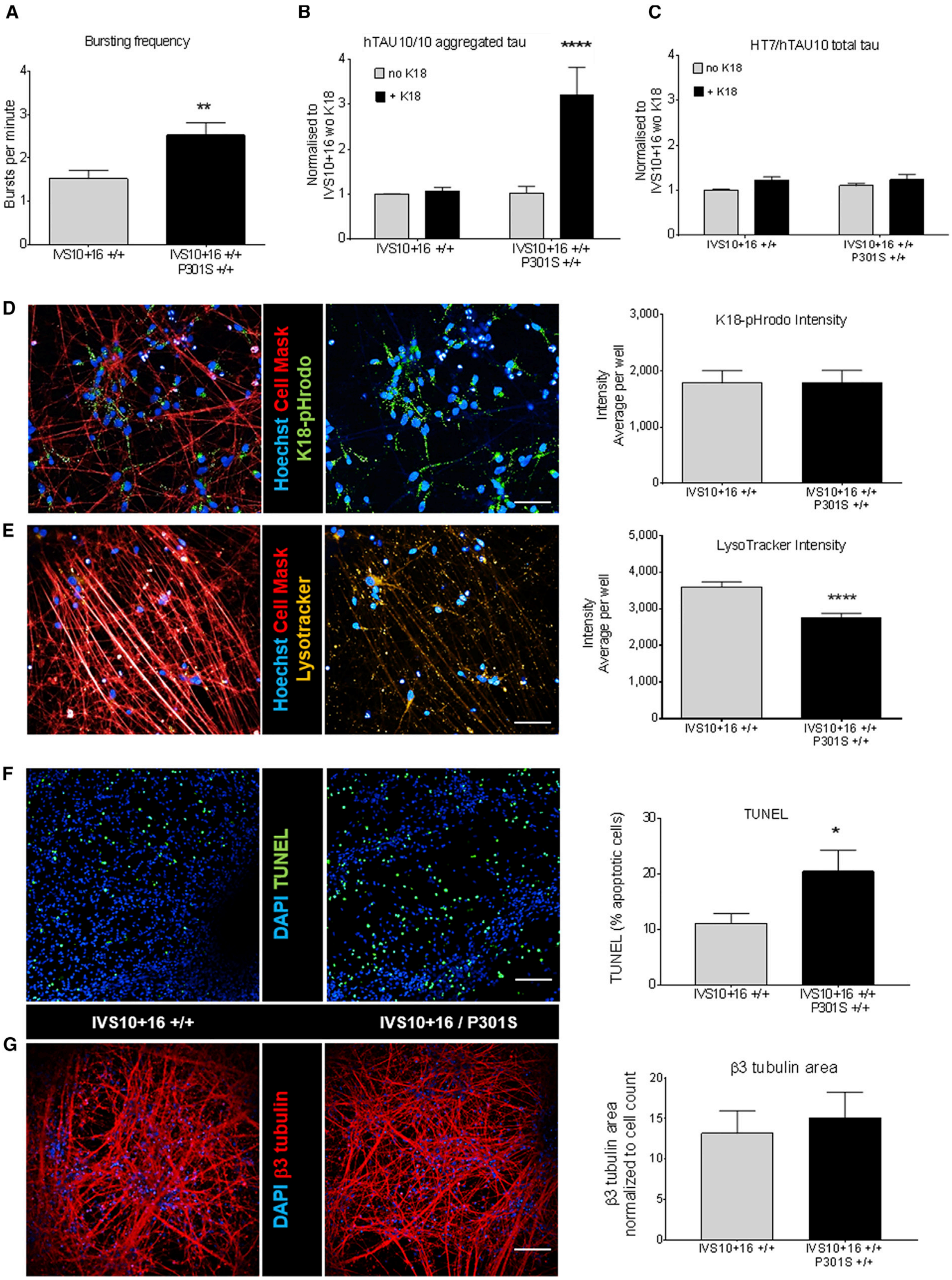

(legend on next page)

functions during development (Sapir et al., 2012). Remarkably, FTDP-17-related progranulin haplo-insufficiency has also been linked with dysregulation of WNT signaling pathways (Rosen et al., 2011; Alquezar et al., 2014) and aberrant cortical differentiation of iPSCs (Raitano et al., 2015).

Accordingly, WNT signaling is indispensable for normal brain development and neural cell fate commitment (Harrison-Uy and Pleasure, 2012) and its link with the IVS10+16 mutation suggests that developmental aberrations might underlie the regional neuronal selectivity and vulnerability that are characteristic for FTDP-17-related tauopathies. Indeed, an aberrant WNT/SHH signaling pathway, as suggested by whole-transcriptome analysis, might cause the observed differences in neuronal cell types in our purely neuronal *in vitro* model and therefore might exacerbate the phenotypes and mechanisms that are potentially linked with the *MAPT* IVS10+16 mutation, as compensatory factors and additional cell types and cellular interactions are lacking in our current model. Also, analysis on postmortem IVS10+16 patient brain tissue would be interesting, to explore whether similar affected pathways can be identified as in our *in vitro* model.

We conclude that the set of gene-edited iPSC lines with *MAPT* IVS10+16 and *MAPT* IVS10+16/P301S mutations that we described could serve as a platform to identify new targets for drug development and to reveal the mechanisms underlying FTDP-17 and other tauopathies. All ZFN-engineered iPSC lines are available to the scientific community via EBiSC (https://cells.ebisc.org) and could therefore be used by others to further identify and/or confirm *MAPT* IVS10+16 and/or P301S-related phenotypes in comparison with the appropriate isogenic control.

EXPERIMENTAL PROCEDURES

Human iPSC Culture and Differentiation into Cortical Neurons

Human iPSCs were cultured feeder free and fed daily with fresh mTeSR™1 medium (Stem Cell Technologies). Cells were passaged with EDTA (Gibco) at confluency. Differentiation into NPCs and cortical neurons was performed using an adapted dual SMAD inhibition protocol (Kuijlaars et al., 2016; Shi et al., 2012). More information can be found in the Supplemental Information.

RNA Extraction and RT-qPCR

Cells were lysed with RLT buffer (Qiagen) supplemented with 1% β-mercaptoethanol. RNA extraction was done using the RNeasy mini kit (Qiagen) followed by cDNA preparation using SuperScript III (Life Technologies). More detailed information on qRT-PCR, microarray analysis, and single-cell data analysis can be found in the Supplemental Information. Note that patient 1 and patient 2 in this manuscript refer to iPSC clones V97 and TSM.

Western Blot

Cells were washed gently with PBS and lysed in radioimmunoprecipitation assay (RIPA) buffer (Gibco) supplemented with protease and phosphatase inhibitors (HALT; Invitrogen). Protein was loaded on Bis-Tris gels and, after SDS PAGE, gels were blotted on nitrocellulose and blocked for 1 hr at room temperature (RT) in Tris-buffered saline (TBS) 0.1% Tween 20 supplemented with 5% milk. Detection was done with horseradish peroxidase-labeled secondary antibodies and the West Dura or West Femto enhanced chemiluminescence kit. More information can be found in the Supplemental Information.

Immunocytochemistry

Cells were fixed for 15 min using 4% paraformaldehyde and 4% sucrose in PBS, washed, and permeabilized for 15 min with Triton X-100 (0.25%) in TBS (50 mM TrisHCl,150 mM NaCl, pH 7,5). After 30 min blocking with 5% normal donkey serum in TBS-Triton-X100 (0.25%), cells were incubated overnight in blocking buffer at 4°C with the following primary antibodies: rabbit anti-VGLUT2, rabbit anti-OCT4, mouse anti- ISL1, mouse anti-RD4, mouse anti-NANOG, mouse and rabbit anti-TUBB3, rabbit anti-TTF1, rabbit anti-TBR1, and rat anti-CTIP2. Subsequently, cells were washed with TBS and incubated for 1 hr at RT with Alexa Fluor 488, Alexa Fluor 594, or Alexa Fluor 647 secondary antibodies (Invitrogen). DAPI or Hoechst was used to stain the nuclei. The immunocytochemistry based Click-iT Plus EdU Imaging Kit and TUNEL kit were used as per manufacturer's instructions to quantify the number of proliferating and apoptotic cells, respectively. Confocal

Figure 7. *MAPT* IVS10+16/P301S Neurons Display Early FTDP-17-Related Phenotypes

(A) Live cell calcium imaging using Fluo-4 shows an increased burst frequency in P301S/IVS10+16 mutant neurons (DIV65) compared with IVS10+16 controls (n = 3; $p < 0.01$).

(B and C) AlphaLISA revealing an increased signal for aggregated (hTAU10/hTAU10) tau in P301S mutant neurons (B), only after seeding with K18 (n = 3; $p < 0.0001$), while total tau levels (C) are not affected (HT7/hTAU10) (p = NS).

(D) Live imaging of K18-pHrodo uptake in combination with Cell Mask reveals no differences between control and mutant neurons (DIV65; n = 4; p = NS).

(E) Live imaging using LysoTracker shows a decreased lysosomal intensity in P301S mutant neurons compared with controls (DIV65; n = 4; $p < 0.0001$). Representative images for K18-Phrodo and LysoTracker are shown. Scale bars represent 50 μm.

(F and G) TUNEL staining and β3-tubulin staining showing increased apoptosis in P301S mutant neurons compared with control without affecting the β3-tubulin-positive area (DIV65); n = 3, p = 0.042 for (F); n = 4 and p = 0.10 for (G).

Scale bars represent 100 μm. *$p < 0.05$, **$p < 0.01$, and ****$p < 0.0001$; t test for (A) and (F), Mann-Whitney test for (D), (E), and (G); two-way ANOVA for (B) and (C). See also Figures S5 and S6 and Table S5.

images were taken with Opera Phenix (PerkinElmer) or CV7000 (Yokogawa) high-content imaging readers. Analysis was done using Columbus (PerkinElmer) and Phaedra (in-house developed open-source software available at www.phaedra.io) (Cornelissen et al., 2012). More detailed information about antibodies can be found in the Supplemental Information.

Live Imaging of pHrodo-488 K18 Fibril Uptake and Lysosomes

Pre-aggregated K18 fibrils (40 μM K18:P301L-myc) were labeled with pHrodo Green STP ester (Molecular Probes) per manufacturer's instructions and purified (72 hr) using Slide-A-Lyzer dialysis cassettes (Thermo Fisher Scientific). Neurons were incubated overnight with 75 nM pHrodo-labeled K18 followed by live imaging with Opera Phenix and image analyses using Harmony Software. For visualization of lysosomes, neurons were incubated for 5 min with LysoTracker Red DND-99 (Life Technologies), Hoechst 33342, and CellMask Deep Red Plasma Membrane Stain (both Molecular Probes) followed by live imaging with Opera Phenix and image analyses using Harmony Software. More details on K18 preparation can be found in the Supplemental Information.

Live Cell Calcium Imaging

Cells were loaded for 30 min (37°C and 5% CO_2) with 1 μM Fluo-4-AM in Dulbecco's PBS containing calcium and magnesium and 10 mM glucose, followed by imaging with an inverted confocal laser scanning microscope and analysis using a custom-made MATLAB script. More details can be found in the Supplemental Information.

AlphaLISA

Cells in 96-well plates were lysed in 40 μL/well RIPA buffer with protease and phosphatase inhibitors (Roche). After 20–30 min of gentle shaking at RT, a 5 μL sample was mixed with 20 μL of biotinylated and acceptor-bead-conjugated antibodies in OptiPlate-384 (all Perkin Elmer). After 2 hr of incubation at RT, 25 μL of streptavidin donor beads were added at RT for 30 min followed by detection with the Envision plate reader. Raw values were normalized to transduced control samples (without fibrils) per plate. To allow the detection of aggregates, the monoclonal hTAU10 antibody was conjugated to both acceptor beads and biotin. To measure total tau levels, a biotinylated HT7 antibody was combined with acceptor-bead-conjugated hTAU10 (HT7/hTAU10) (Verheyen et al., 2015).

Statistics

Cells from three independent differentiations were used for all experiments. All data were acquired from at least three independent experiments (n), unless specified otherwise. Data are shown as the mean with SEM. Student's t test was used to compare two groups and one-way or two-way ANOVA was used to compare more than two groups (GraphPad Prism 6). Dunnet's or Sidak's post hoc test was used for multiple comparisons. When the data were not normally distributed or with unequal variance, non-parametric tests were used: a Mann-Whitney test for two groups and a Kruskal-Wallis test with Dunn's post hoc test for more than two groups. p values < 0.05 were considered significantly different.

ACCESSION NUMBERS

All microarray data have been uploaded to GEO: GSE106076.

SUPPLEMENTAL INFORMATION

Supplemental Information includes Supplemental Experimental Procedures, six figures, and six tables and can be found with this article online at https://doi.org/10.1016/j.stemcr.2018.06.022.

AUTHOR CONTRIBUTIONS

A.V., A.D., I.V.d.W., R.D.H., J.K., L.D.M., C.v.O.d'Y., A. Bretteville, A. Buist, A.C.-S., S.W., and I.R. designed and performed the experiments. A.V., J.R., K.V.H., R.D.H., A.D.B., J.K., L.D.M., C.v.O.d'Y., A. Bretteville, and S.J. analyzed the data. A.V. wrote the manuscript with the help of A.E., P.R., I.R., and P.J.P.

ACKNOWLEDGMENTS

The research leading to these results has received support from the Innovative Medicines Initiative Joint Undertaking under grant agreements number 115439 (StemBANCC) and 115582 (EBiSC), resources of which are composed of financial contributions from the European Union's Seventh Framework Program (FP7/2007-2013) and EFPIA companies' in-kind contribution. This publication reflects only the author's views, and neither the IMI JU nor EFPIA nor the European Commission is liable for any use that may be made of the information contained therein. We would like to thank Jason Gustin and Mark Gerber from Sigma-Aldrich for the generation of all gene-edited (ZFN) *MAPT* lines and Sjors Den Boer for technical assistance. Authors A.V., A.D., J.R., I.V.d.W., C.v.O.d'Y., A.D.B., L.D.M., R.D.H., A. Bretteville, S.J., A. Buist, A.C.S., A.E., P.R., I.R., and P.J.P. are employees of Janssen Pharmaceutica N.V.

Received: September 30, 2017
Revised: June 29, 2018
Accepted: June 29, 2018
Published: July 26, 2018

REFERENCES

Alquezar, C., Esteras, N., De La Encarnacion, A., Alzualde, A., Moreno, F., Lopez De Munain, A., and Martin-Requero, A. (2014). PGRN haploinsufficiency increased Wnt5a signaling in peripheral cells from frontotemporal lobar degeneration-progranulin mutation carriers. Neurobiol. Aging *35*, 886–898.

Baba, Y., Baker, M.C., Le Ber, I., Brice, A., Maeck, L., Kohlhase, J., Yasuda, M., Stoppe, G., Bugiani, O., Sperfeld, A.D., et al. (2007). Clinical and genetic features of families with frontotemporal dementia and parkinsonism linked to chromosome 17 with a P301S tau mutation. J. Neural Transm. (Vienna) *114*, 947–950.

Beevers, J.E., Lai, M.C., Collins, E., Booth, H.D.E., Zambon, F., Parkkinen, L., Vowles, J., Cowley, S.A., Wade-Martins, R., and Caffrey, T.M. (2017). MAPT genetic variation and neuronal maturity alter isoform expression affecting axonal transport in iPSC-derived dopamine neurons. Stem Cell Reports *9*.

Brun, A., Englund, B., Gustafson, L., Passant, U., Mann, D.M.A., Neary, D., and Snowden, J.S. (1994). Clinical and neuropathological

criteria for frontotemporal dementia. The Lund and Manchester Groups. J. Neurol. Neurosurg. Psychiatry *57*, 416–418.

Bugiani, O., Murrell, J.R., Giaccone, G., Hasegawa, M., Ghigo, G., Tabaton, M., Morbin, M., Primavera, A., Carella, F., Solaro, C., et al. (1999). Frontotemporal dementia and corticobasal degeneration in a family with a P301S mutation in tau. J. Neuropathol. Exp. Neurol. *58*, 667–677.

Chen, S., Townsend, K., Goldberg, T.E., Davies, P., and Conejero-Goldberg, C. (2010). MAPT isoforms: differential transcriptional profiles related to 3R and 4R splice variants. J. Alzheimers Dis. *22*, 1313–1329.

Cornelissen, F., Cik, M., and Gustin, E. (2012). Phaedra, a protocol-driven system for analysis and validation of high-content imaging and flow cytometry. J. Biomol. Screen. *17*, 496–506.

Drechsel, D.N., Hyman, A.A., Cobb, M.H., and Kirschner, M.W. (1992). Modulation of the dynamic instability of tubulin assembly by the microtubule-associated protein tau. Mol. Biol. Cell *3*, 1141–1154.

Fernandez-Nogales, M., Cabrera, J.R., Santos-Galindo, M., Hoozemans, J.J., Ferrer, I., Rozemuller, A.J., Hernandez, F., Avila, J., and Lucas, J.J. (2014). Huntington's disease is a four-repeat tauopathy with tau nuclear rods. Nat. Med. *20*, 881–885.

Fernandez-Nogales, M., Santos-Galindo, M., Hernandez, I.H., Cabrera, J.R., and Lucas, J.J. (2016). Faulty splicing and cytoskeleton abnormalities in Huntington's disease. Brain Pathol. *26*, 772–778.

Garcia-Cabrero, M., Guerrero-Lopez, R., Giraldez, B.G., Llorens-Martin, M., Avila, J., Serratosa, J.M., and Sanchez, M.P. (2013). Hyperexcitability and epileptic seizures in a model of frontotemporal dementia. Neurobiol. Dis. *58*, 200–208.

Geschwind, H., Robidoux, J., Alarcon, M., Miller, B.L., Wilhelmsen, K.C., Cummings, J.L., and Nasreddine, Z.S. (2001). Dementia and neurodevelopmental predisposition: cognitive dysfunction in presymptomatic subjects precedes dementia by decades in frontotemporal dementia. Ann. Neurol. *50*, 741–746.

Ghetti, B., Oblak, A.L., Boeve, B.F., Johnson, B.C., Dickerson, K.A., and Goedert, M. (2015). Invited review: frontotemporal dementia caused by microtubule-associated protein tau gene (MAPT) mutations: a chameleon for neuropathology and neuroimaging. Neuropathol. Appl. Neurobiol. *41*, 24–46.

Grover, A., Houlden, H., Baker, M., Adamson, J., Lewis, J., Prihar, G., Pickering-Brown, S., Duff, K., and Hutton, M. (1999). 5′ splice site mutations in tau associated with the inherited dementia FTDP-17 affect a stem-loop structure that regulates alternative splicing of exon 10. J. Biol. Chem. *274*, 15134–15143.

Guo, J.L., and Lee, V.M. (2013). Neurofibrillary tangle-like tau pathology induced by synthetic tau fibrils in primary neurons over-expressing mutant tau. FEBS Lett. *587*, 717–723.

Guo, J.L., Buist, A., Soares, A., Callaerts, K., Calafate, S., Stevenaert, F., Daniels, J.P., Zoll, B.E., Crowe, A., Brunden, K.R., et al. (2016). The dynamics and turnover of tau aggregates in cultured cells: insights into therapies for tauopathies. J. Biol. Chem. *291*, 13175–13193.

Harrison-Uy, S.J., and Pleasure, S.J. (2012). Wnt signaling and forebrain development. Cold Spring Harb. Perspect. Biol. *4*, a008094.

Hutton, M., Lendon, C.L., Rizzu, P., Baker, M., Froelich, S., Houlden, H., Pickering-Brown, S., Chakraverty, S., Isaacs, A., Grover, A., et al. (1998). Association of missense and 5′-splice-site mutations in tau with the inherited dementia FTDP-17. Nature *393*, 702–705.

Imamura, K., Sahara, N., Kanaan, N.M., Tsukita, K., Kondo, T., Kutoku, Y., Ohsawa, Y., Sunada, Y., Kawakami, K., Hotta, A., et al. (2016). Calcium dysregulation contributes to neurodegeneration in FTLD patient iPSC-derived neurons. Sci. Rep. *6*, 34904.

Iovino, M., Agathou, S., Gonzalez-Rueda, A., Del Castillo Velasco-Herrera, M., Borroni, B., Alberici, A., Lynch, T., O'Dowd, S., Geti, I., Gaffney, D., et al. (2015). Early maturation and distinct tau pathology in induced pluripotent stem cell-derived neurons from patients with MAPT mutations. Brain *138*, 3345–3359.

Kanaan, N.M., Morfini, G.A., Lapointe, N.E., Pigino, G.F., Patterson, K.R., Song, Y., Andreadis, A., Fu, Y., Brady, S.T., and Binder, L.I. (2011). Pathogenic forms of tau inhibit kinesin-dependent axonal transport through a mechanism involving activation of axonal phosphotransferases. J. Neurosci. *31*, 9858–9868.

Korade, Z., and Mirnics, K. (2011). Wnt signaling as a potential therapeutic target for frontotemporal dementia. Neuron *71*, 955–957.

Kosik, K.S., Orecchio, L.D., Bakalis, S., and Neve, R.L. (1989). Developmentally regulated expression of specific tau sequences. Neuron *2*, 1389–1397.

Kuijlaars, J., Oyelami, T., Diels, A., Rohrbacher, J., Versweyveld, S., Meneghello, G., Tuefferd, M., Verstraelen, P., Detrez, J.R., Verschuuren, M., et al. (2016). Sustained synchronized neuronal network activity in a human astrocyte co-culture system. Sci. Rep. *6*, 36529.

Lake, B., Ai, R., Kaeser, G.E., Salathia, N.S., Yung, Y.C., Liu, R., Wildberg, A., Gao, D., Fung, H.L., Chen, S., et al. (2016). Neuronal subtypes and diversity revealed by single-nucleus RNA sequencing of the human brain. Science *352*, 1586–1590.

Lopez-Gonzalez, I., Aso, E., Carmona, M., Armand-Ugon, M., Blanco, R., Naudi, A., Cabre, R., Portero-Otin, M., Pamplona, R., and Ferrer, I. (2015). Neuroinflammatory gene regulation, mitochondrial function, oxidative stress, and brain lipid modifications with disease progression in tau P301S transgenic mice as a model of frontotemporal lobar degeneration-tau. J. Neuropathol. Exp. Neurol. *74*, 975–999.

Majounie, E., Cross, W., Newsway, V., Dillman, A., Vandrovcova, J., Morris, C.M., Nalls, M.A., Ferrucci, L., Owen, M.J., O'Donovan, M.C., et al. (2013). Variation in tau isoform expression in different brain regions and disease states. Neurobiol. Aging *34*, 1922.e7–1922.e12.

Malmanche, N., Dourlen, P., Gistelinck, M., Demiautte, F., Link, N., Dupont, C., Vanden Broeck, L., Werkmeister, E., Amouyel, P., Bongiovanni, A., et al. (2017). Developmental expression of 4-repeat-tau induces neuronal aneuploidy in *Drosophila* tauopathy models. Sci. Rep. *7*, 40764.

Medda, X., Mertens, L., Versweyveld, S., Diels, A., Barnham, L., Bretteville, A., Buist, A., Verheyen, A., Royaux, I., Ebneth, A., et al. (2016). Development of a scalable, high-throughput-compatible assay to detect tau aggregates using iPSC-derived cortical

neurons maintained in a three-dimensional culture format. J. Biomol. Screen. *21*, 804–815.

Park, S.A., Ahn, S.I., and Gallo, J.M. (2016). Tau mis-splicing in the pathogenesis of neurodegenerative disorders. BMB Rep. *49*, 405–413.

Raitano, S., Ordovas, L., De Muynck, L., Guo, W., Espuny-Camacho, I., Geraerts, M., Khurana, S., Vanuytsel, K., Toth, B.I., Voets, T., et al. (2015). Restoration of progranulin expression rescues cortical neuron generation in an induced pluripotent stem cell model of frontotemporal dementia. Stem Cell Rep. *4*, 16–24.

Riise, J., Plath, N., Pakkenberg, B., and Parachikova Aberrant, A. (2015). Wnt signaling pathway in medial temporal lobe structures of Alzheimer's disease. J. Neural Transm. (Vienna) *122*, 1303–1318.

Rosen, E.Y., Wexler, E.M., Versano, R., Coppola, G., Gao, F., Winden, K.D., Oldham, M.C., Martens, L.H., Zhou, P., Farese, R.V., Jr., et al. (2011). Functional genomic analyses identify pathways dysregulated by progranulin deficiency, implicating Wnt signaling. Neuron *71*, 1030–1042.

Sapir, T., Frotscher, M., Levy, T., Mandelkow, E.M., and Reiner, O. (2012). Tau's role in the developing brain: implications for intellectual disability. Hum. Mol. Genet. *21*, 1681–1692.

Sennvik, K., Boekhoorn, K., Lasrado, R., Terwel, D., Verhaeghe, S., Korr, H., Schmitz, C., Tomiyama, T., Mori, H., Krugers, H., et al. (2007). Tau-4R suppresses proliferation and promotes neuronal differentiation in the hippocampus of tau knockin/knockout mice. FASEB J. *21*, 2149–2161.

Shi, Y., Kirwan, P., and Livesey, F.J. (2012). Directed differentiation of human pluripotent stem cells to cerebral cortex neurons and neural networks. Nat. Protoc. *7*, 1836–1846.

Sperfeld, D., Collatz, M.B., Baier, H., Palmbach, M., Storch, A., Schwarz, J., Tatsch, K., Reske, S., Joosse, M., Heutink, P., et al. (1999). FTDP-17: an early-onset phenotype with parkinsonism and epileptic seizures caused by a novel mutation. Ann. Neurol. *46*, 708–715.

Spillantini, M.G., and Goedert, M. (2013). Tau pathology and neurodegeneration. Lancet Neurol. *12*, 609–622.

Sposito, T., Preza, E., Mahoney, C.J., Seto-Salvia, N., Ryan, N.S., Morris, H.R., Arber, C., Devine, M.J., Houlden, H., Warner, T.T., et al. (2015). Developmental regulation of tau splicing is disrupted in stem cell-derived neurons from frontotemporal dementia patients with the 10 + 16 splice-site mutation in MAPT. Hum. Mol. Genet. *24*, 5260–5269.

Verheyen, A., Diels, A., Dijkmans, J., Oyelami, T., Meneghello, G., Mertens, L., Versweyveld, S., Borgers, M., Buist, A., Peeters, P., et al. (2015). Using human iPSC-derived neurons to model tau aggregation. PLoS One *10*, e0146127.

Whitwell, L., Jack, C.R., Jr., Boeve, B.F., Senjem, M.L., Baker, M., Ivnik, R.J., Knopman, D.S., Wszolek, Z.K., Petersen, R.C., Rademakers, R., et al. (2009). Atrophy patterns in IVS10+16, IVS10+3, N279K, S305N, P301L, and V337M MAPT mutations. Neurology *73*, 1058–1065.

Xu, Y., Martini-Stoica, H., and Zheng, H. (2016). A seeding based cellular assay of tauopathy. Mol. Neurodegener. *11*, 32.

Yoshiyama, Y., Higuchi, M., Zhang, B., Huang, S.M., Iwata, N., Saido, T.C., Maeda, J., Suhara, T., Trojanowski, J.Q., and Lee, V.M. (2007). Synapse loss and microglial activation precede tangles in a P301S tauopathy mouse model. Neuron *53*, 337–351.

Stem Cell Reports, Volume *11*

Supplemental Information

Genetically Engineered iPSC-Derived FTDP-17 *MAPT* Neurons Display Mutation-Specific Neurodegenerative and Neurodevelopmental Phenotypes

An Verheyen, Annick Diels, Joke Reumers, Kirsten Van Hoorde, Ilse Van den Wyngaert, Constantin van Outryve d'Ydewalle, An De Bondt, Jacobine Kuijlaars, Louis De Muynck, Ronald De Hoogt, Alexis Bretteville, Steffen Jaensch, Arjan Buist, Alfredo Cabrera-Socorro, Selina Wray, Andreas Ebneth, Peter Roevens, Ines Royaux, and Pieter J. Peeters

Supplemental Figures

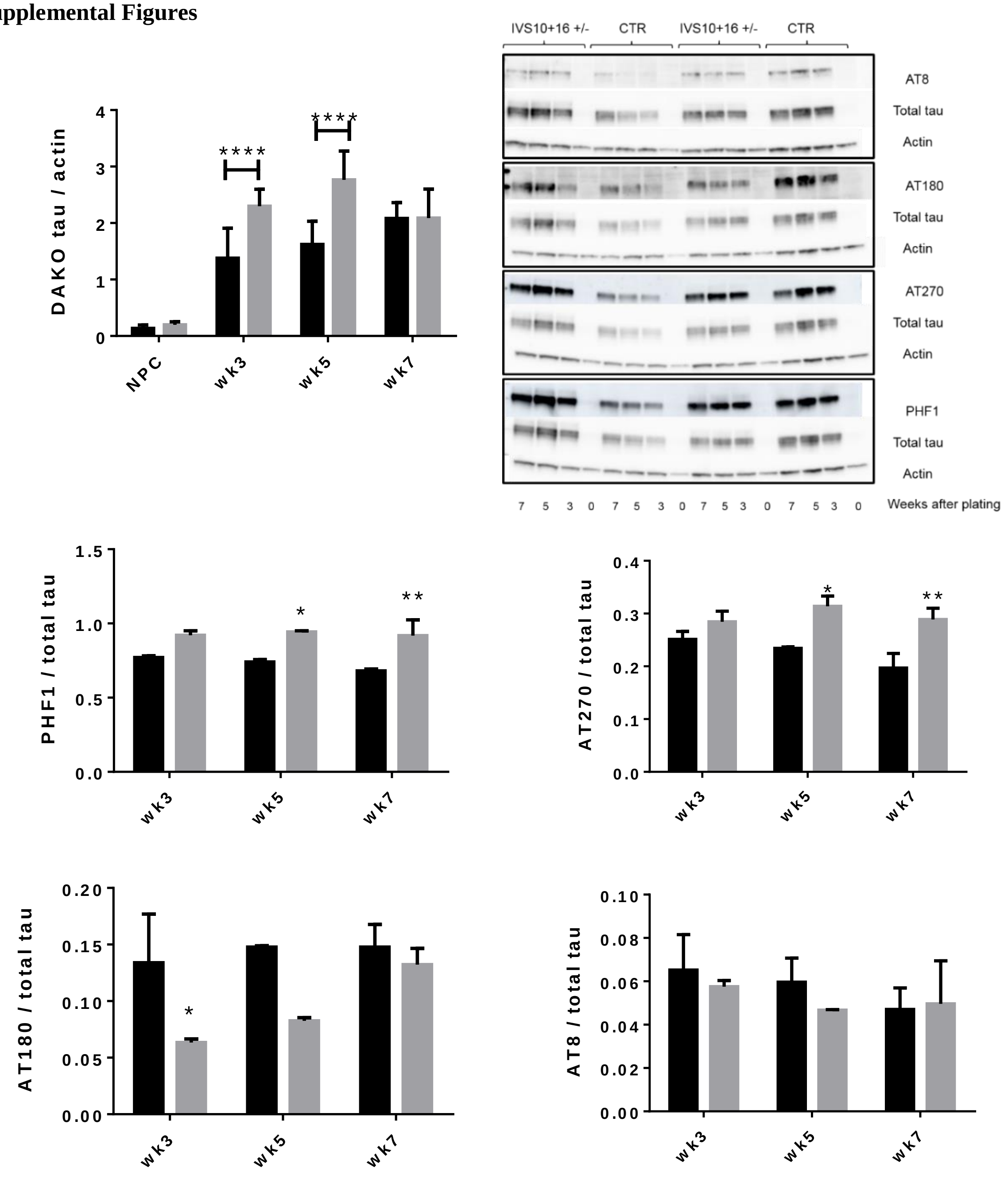

Figure S1: Western blot time course of total tau and phosphorylated tau on control and IVS10+16 +/- neurons. Related to Figure 2. Western blot for total tau and phospho-tau (DAKO total tau, PHF1, AT8, AT270 and AT180; all 1μg/ml). Duplo's from 2 independent differentiations are shown. Phospho tau blots were re-probed with total tau antibodies for quantification. Total tau levels are increased over time in control and mutant neurons ($P<0.0001$, n=4, NPC *versus* wk3 (DIV51), wk5 (DIV65) and wk7 (DIV80) after plating for both control and IVS10+16 cells), with also more total tau in neurons carrying the IVS10+16 mutation at 3 and 5 weeks after plating (n=4; $P<0.0001$). There is an increased phosphorylation at tau Ser396/Ser404 (PHF1) and Thr181 (AT270) in IVS10+16 carrying neurons 5 and 7 weeks after plating while there is less phosphorylation at Thr231 (AT180) 3 weeks after plating; *$P<0.05$, **$P<0.01$ and ****$P<0.0001$ (2-way ANOVA).

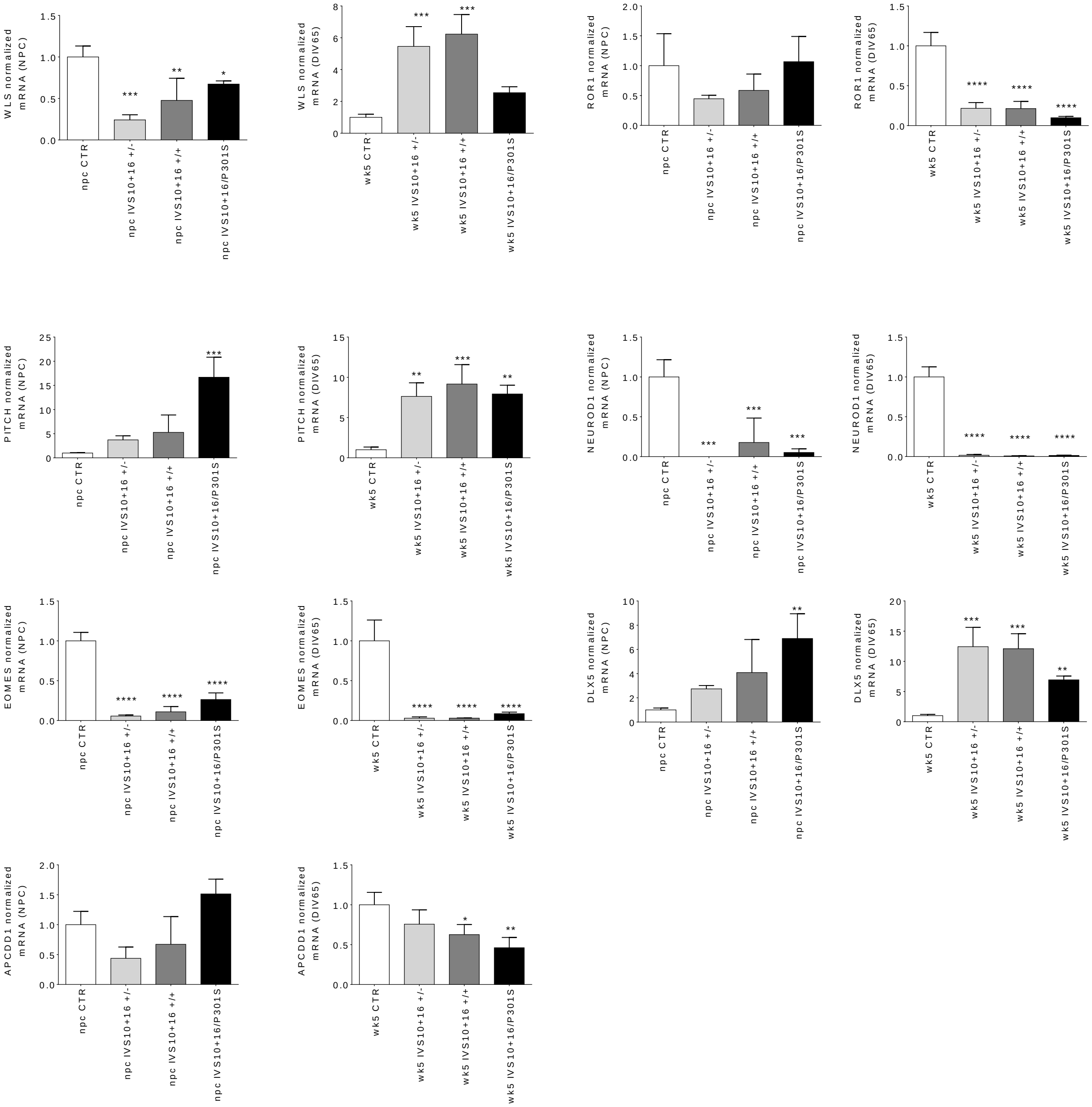

Figure S2: RT-qPCR confirmation of selected genes in control and mutant NPCs and neurons. Related to Figure 3 and Tables S1 and S2. Cells were lysed at DIV31 (NPC) or DIV65 (week 5). *P<0.05, **P<0.01, ***P<0.001 and ****P<0.0001 (1-way ANOVA, n=3).

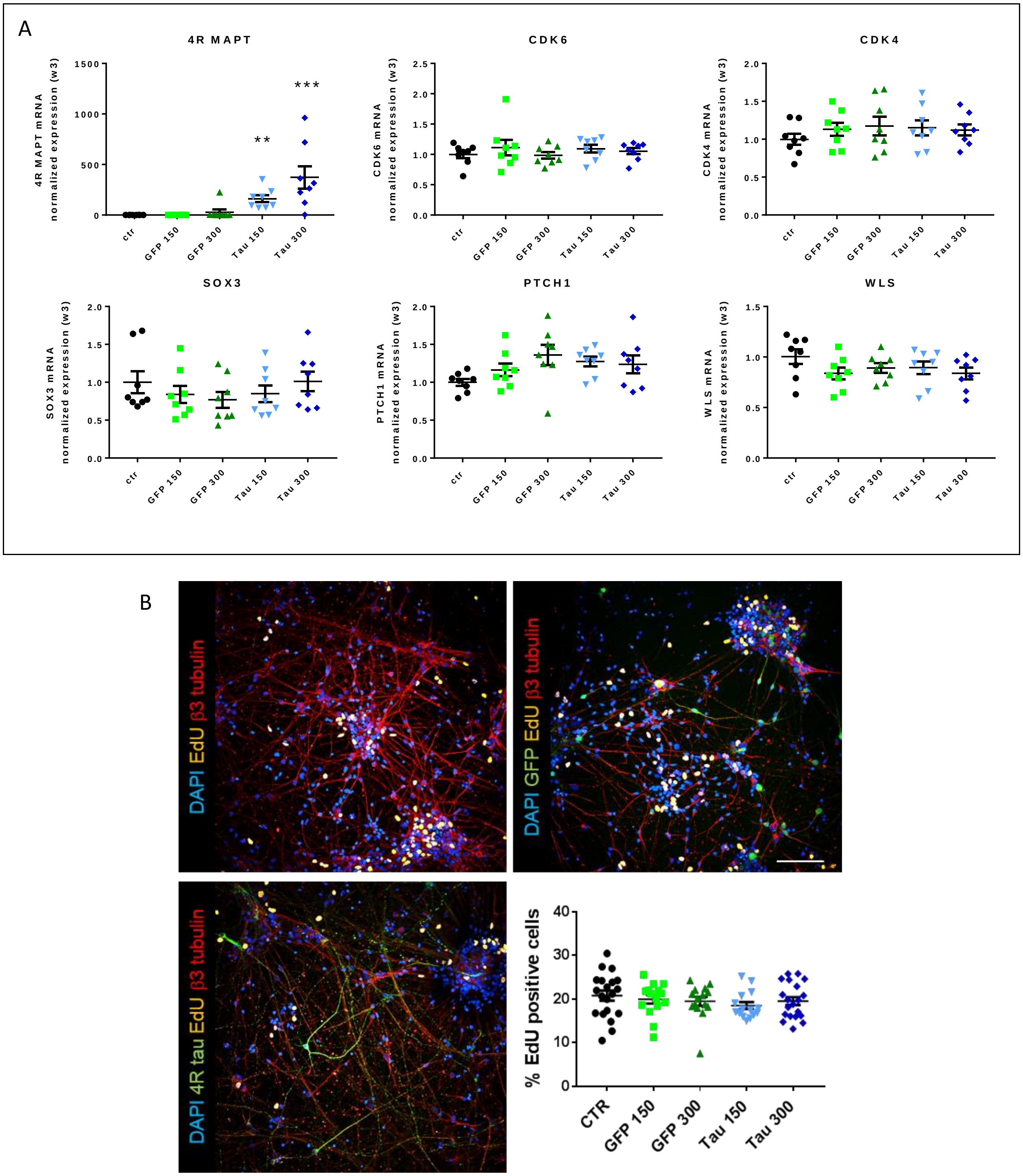

Figure S3: AAV6 induced 4R tau overexpression in control cortical neurons does not recapitulate IVS10+16-induced phenotypes. Related to Figures 3-4 and Figure S2 (A) RT-qPCR on selected genes from cell cycle and SHH/WNT pathways (4R MAPT; $P<0.0001$, n=3 and for all other genes P=NS, n=3). MOI of 150 or 300 was used. The wild type 2N4R tau isoform was used to overexpress 4R tau **$P<0.01$ and ***$P<0.001$ (Kruskal Wallis test for 4R MAPT and 1-way ANOVA for all other genes). (B) EdU staining 3 weeks after transduction with AAV (P=NS, n=3, Kruskal-Wallis test). Representative pictures are shown of control, 4R tau-transduced or GFP-transduced neurons. Scale bar represents 100µm.

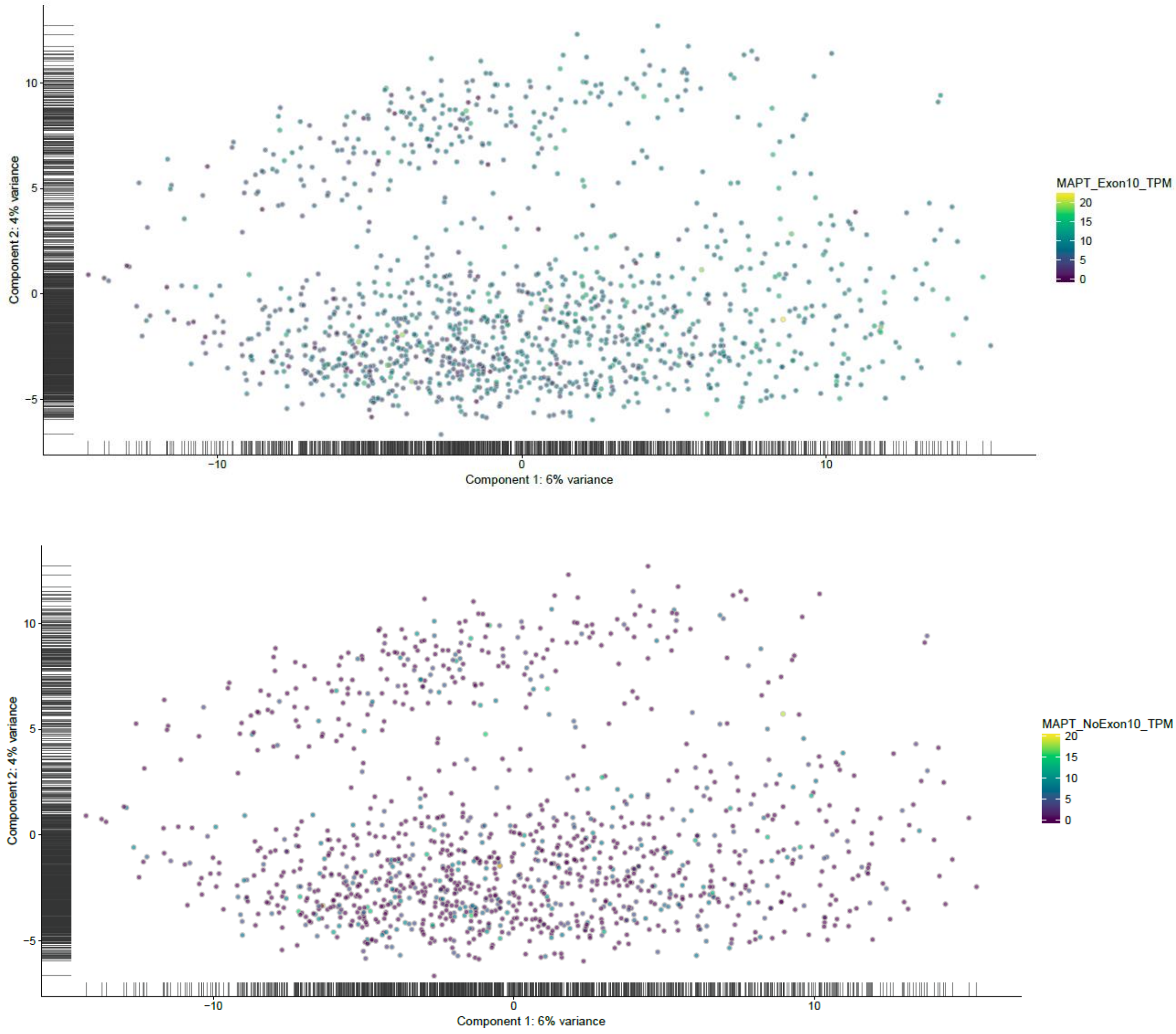

Figure S4: Heterogeneous expression of *MAPT* with and without exon 10 after single cell RNA sequencing of human cerebral cortex tissue. Related to Figure 5. Color mapping of *MAPT* with exon 10 (MAPT_exon10) and *MAPT* without exon 10 (MAPT_no_ex10) reveals a heterogeneous but overall higher expression profile of MAPT_ex10 than MAPT_no_exon10, without clustering of cell populations.

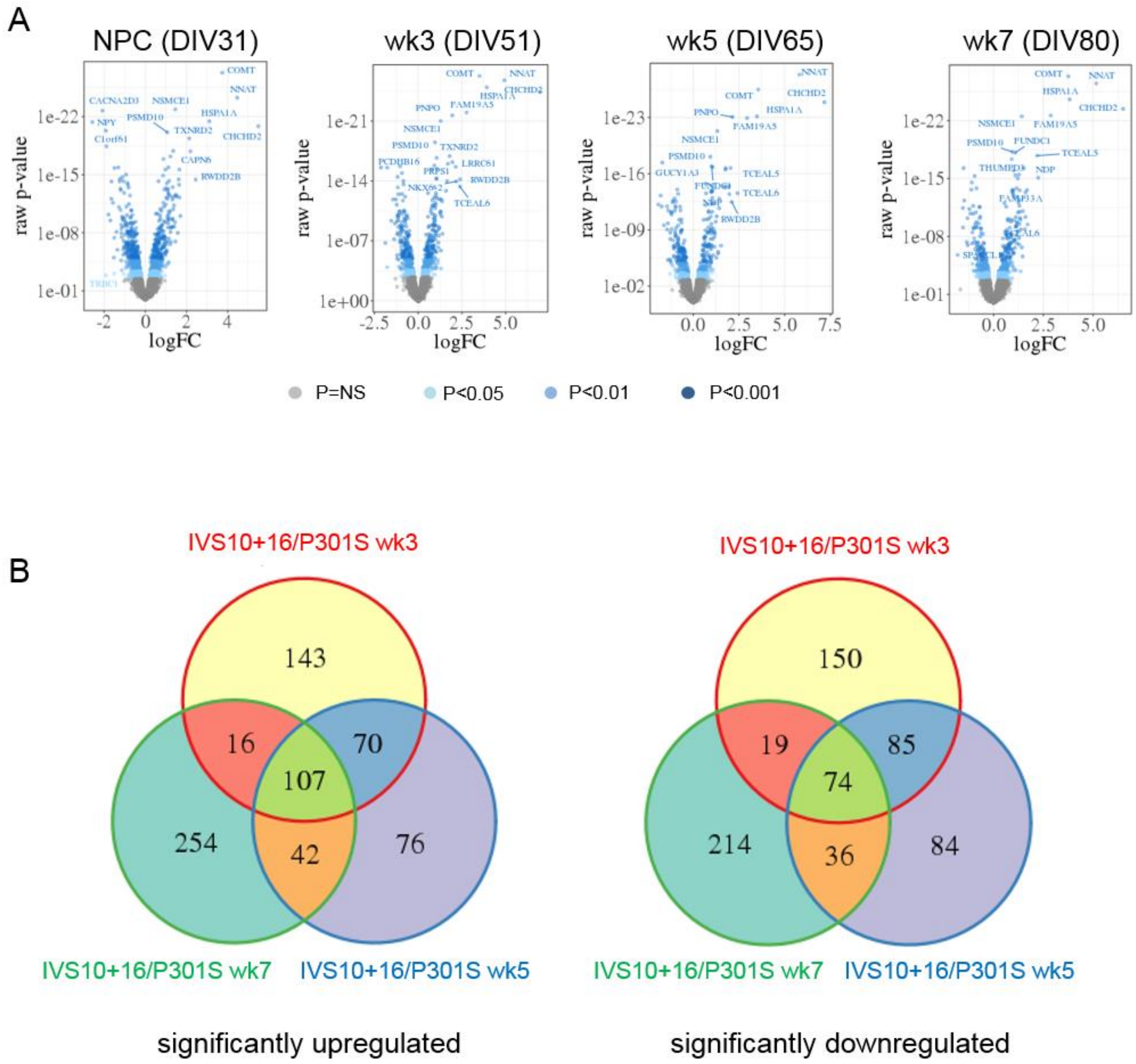

Figure S5: Microarray analysis on IVS10+16 / P301S versus IVS10+16 neurons at different time points. Related to Figure 7. (A) Volcano plots on time points NPC, week 3, week 5 and week 7 after plating. The top genes with the highest significance and/or highest fold change are highlighted. (B) Venn diagrams showing overlapping genes between 3 different neuronal timepoints (3, 5 and 7 weeks after plating) when comparing IVS10+16/P301S neurons to their respective controls (IVS10+16 +/+). 107 genes are significantly upregulated and 74 genes are significantly downregulated at all timepoints.

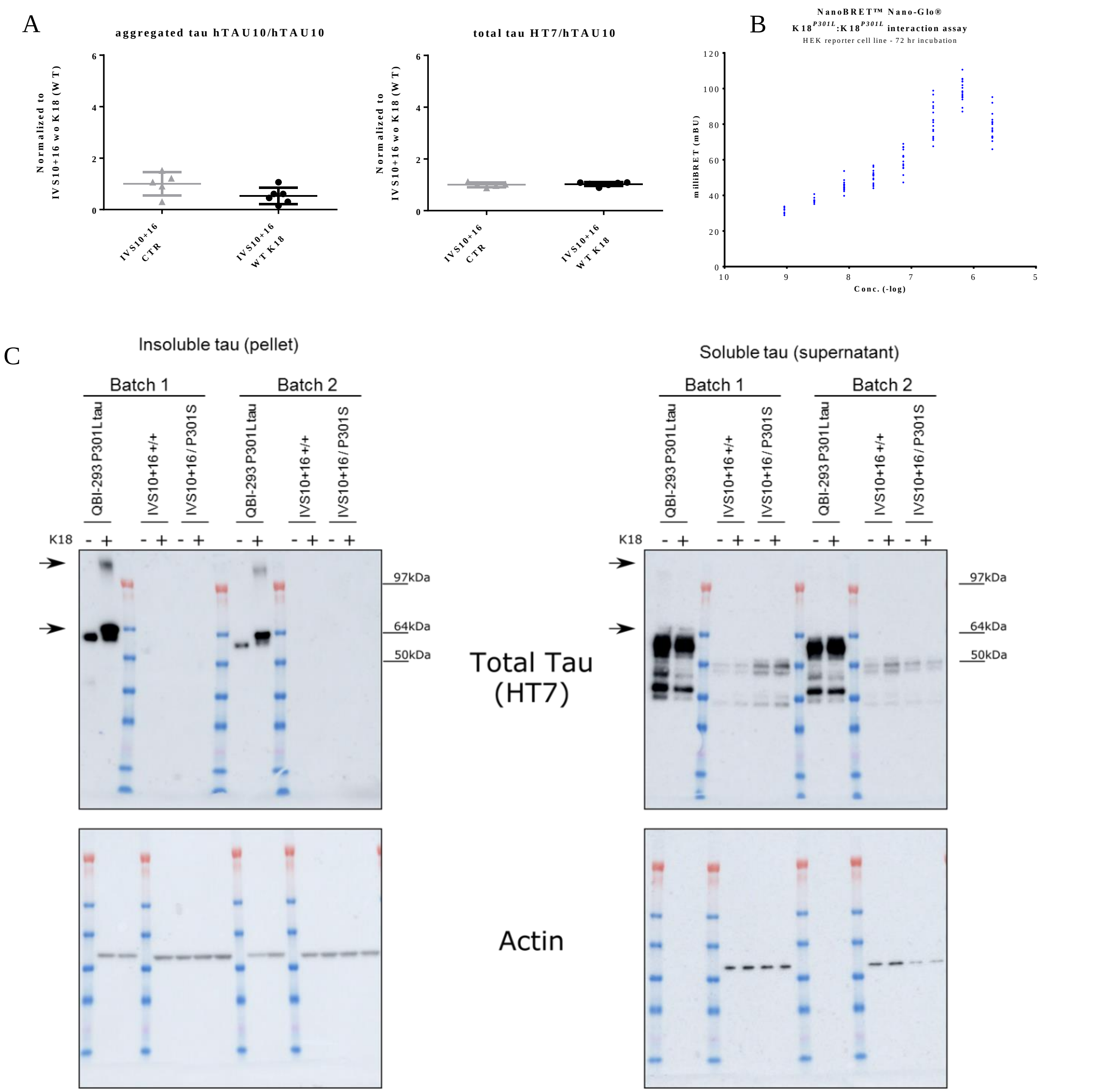

Figure S6: Seeding-potent wild type K18 does not induce aggregation in single mutant IVS10+16 neurons and P301L-K18 does not induce insoluble tau. Related to Figure 7. (A) AlphaLISA for aggregated tau (hTAU10/hTAU10) and total tau shows no difference in signal after addition of wild type K18 seeds (n=3 experiments, P=NS for both, T-test). (B) The NanoBret™Nano-Glo® K18:K18 interaction assay using a HEK reporter cell line (with overexpression of P301L tau) shows that wild type K18 induces a robust, concentration-dependent BRET signal corresponding to tau aggregation after 72 hours of K18-WT incubation. (C) Sarkosyl extraction of insoluble tau from iPSCs derived neurons, with and without K18P301L (lysis 7 weeks after seeding)**.** Equal amounts of protein from iPSC neurons were subjected to Sarkosyl extraction of insoluble material. Insoluble pellets and supernatants were then immunoblotted for total human tau antibody (HT7). Actin immunoblotting of supernatant is used as loading control. Two separate extractions are represented. Lysate of QBI293 cells transiently transfected with P301L tau and seeded with K18 fibrils (dilution 1/30 compared to the other samples), is used in each extraction batch as a positive control of successful extraction of insoluble tau.

Table S1: Differential expression of genes related to neuronal subtypes and cell cycle regulation due to IVS10+16 mutation. Related to Figures 3 and 4.

**Significantly downregulated forebrain genes (logFC) IVS10+16 *vs* control**	**Significantly upregulated interneuron/basal ganglia/limbic genes (logFC) IVS10+16 *vs* control**	**Significantly different transcription factors/ cell cycle regulators (logFC) IVS10+16 *vs* control**
*EOMES (-5.00) *** **	*ISL1 (4.21) *** **	*TCF3 (-0.39) ***
*TBR1 (-3.78) *** **	*RELN (4.07) *** **	*TCF4 (-0.87) *** **
*SATB2 (-1.85) *** **	*DLX6 (3.89) *** **	*HEY2 (-1.22) *** **
*NEUROD1 (-4.63) *** **	*DLX2 (2.80) *** **	*SOX3 (-2.64) *** **
*NEUROD2 (-5.46) *** **	*DLX1 (2.72) *** **	*ZFPM2 (-4.63) *** **
*NEUROD6 (-7.25) *** **	*DLX5 (3.05) *** **	*CDK2 (-0.70) *** **
*EMX1 (-2.61) *** **	*GAD2 (2.45) *** **	*CDK4 (-1.03) *** **
*EMX2 (-2.95) *** **	*GAD1 (2.31) *** **	*CDK6 (2.64) *** **
*SLC17A7 (-3.86) *** **	*SLC32A1 (3.29) *** **	*CDK17 (0.63) *** **
*SLC17A6 (-4.22) *** **	*LHX6 (2.51) *** **	*CDK18 (-0.58) *** **
*LHX2 (-2.28) *** **	*LHX8 (3.64) *** **	*CDK19 (0.48) ***
*FEZF2 (-6.02) *** **	*GSX2 (2.30) *** **	*CDK20 (0.71) ***
*NEUROG2 (-5.69) *** **	*LSAMP (1.67) *** **	*p18 CDKN2C (1.06) *** **
*NEUROG1 (-2.25) *** **	*TH (1.17) *** **	*p19 CDKN2D (0.62) *** **
*LEF1 (-2.77) *** **	*DRD1 (1.48) *** **	*p21 CDKN1A (-1.63) *** **
*HES1 (-2.26) *** **	*DRD2 (0.92) *** **	*p27 CDKN1B (-0.46) ***

Table S1: List of differentially expressed genes after microarray on ZFN monoallelic *MAPT* IVS10+16 neurons five weeks after final plating and compared to the isogenic control. A selection of genes related to forebrain, interneuron/basal ganglia, limbic system and transcription factors/cell cycle regulators is shown. LogFC (fold changes) are shown. **P<0.01; ***P<0.001 and ****P<0.0001. See also Figures 3 and 4.

Table S2: Differential expression of genes related to WNT and SHH signaling at NPC and neuronal stage due to IVS10+16 mutation. Related to Figure 6.

WNT/SHH signaling significant GENES	Time point	Sigma IVS10+16 DIV65 (logFC)	Patient 1 IVS10+16 DIV65 (logFC)	Patient 2 IVS10+16 DIV65 (logFC)
WLS	***DIV65***<br>***NPC***	↑ (2.05) ****<br>↓ (-1.88) ****	↑ (1.21) ****<br>↓ (-2.53) ****	↑ (2.07) ****
WNT4	***DIV65***	↑ (0.51) ****	↑ (0.84) ****	↑ (0.27) *
WNT7A	***DIV65***<br>***NPC***	-<br>↑ (0.78) ****	-<br>-	↑ (0.60) ***<br>↑ (1.38) ****
WNT7B	***DIV65***<br>***NPC***	↓ (-0.30) ***<br>↓ (-0.35) ***	↓ (-0.37) ****<br>↓ (-0.44) ****	↓ (-0.30) ***<br>↓ (-0.34) ***
WNT5B	***DIV65***<br>***NPC***	↓ (-0.34) *<br>-	↓ (-0.54) ***<br>↑ (0.73) ***	↓ (-0.72) ****<br>↑ (0.76) ***
GSK3A	***DIV65***<br>***NPC***	↑ (0.20) *<br>-	↑ (0.21) **<br>↓ (-0.21) *	-<br>-
GSK3B	***DIV65***	↑ (0.27) ***	↑ (0.52) ****	↑ (0.66) ****
AXIN2	***DIV65***	↑ (0.29) **	↑ (0.46) ****	↑ (0.28) **
TCF7L2	***DIV65***<br>***NPC***	-<br>↑ (0.56) ****	↑ (0.20) *<br>↑ (0.40) ***	↑ (0.36) ***<br>-
TCF7L1	***DIV65***<br>***NPC***	↓ (-0.87) ****<br>-	↓ (-0.72) ****<br>↑ (0.43) *	↓ (-0.74) ****<br>-
FZD8	***DIV65***<br>***NPC***	↓ (-0.62) ****<br>-	↓ (-0.46) ****<br>↑ (0.38) ***	↓ (-0.67) ****<br>-
FZD7	***DIV65***<br>***NPC***	↓ (-1.55) ****<br>↓ (-0.85) ****	↓ (-1.97) ****<br>↓ (-1.01) ****	↓ (-1.89) ****<br>↑ (0.69) ****
FZD3	***DIV65***<br>***NPC***	↑ (0.73) ****	↑ (1.30) ****<br>↑ (0.45) ***	↑ (1.06) ****
FZD5	***DIV65***<br>***NPC***	↑ (0.28) *<br>↑ (1.65) ****	↑ (0.93) ****<br>↑ (2.28) ****	-<br>↑ (1.22) ****
FZD2	***DIV65***<br>***NPC***	↓ (-0.81) ****	↓ (-0.66) ****<br>↑ (0.48) ***	↓ (-0.29) *
FZD9	***DIV65***<br>***NPC***	↓ (-0.36) *	-	↓ (-0.42) **<br>↑ (0.51) **
FZD6	***DIV65***<br>***NPC***	↓ (-0.81) ****<br>↓ (-0.46) **	-	↓ (-0.33) **
NIN	***DIV65***<br>***NPC***	↑ (0.50) ****	↑ (0.72) ****<br>↑ (0.49) ****	↑ (1.29) ****
SRFP1	***DIV65***<br>***NPC***	↓ (-0.43) **<br>↑ (0.30) *	↓ (-0.36) **<br>↑ (0.90) ****	-<br>↑ (0.54) ***
LRP2	***DIV65***<br>***NPC***	-<br>↑ (0.90) ****	-<br>↑ (0.92) ****	↑ (0.29) *<br>↑ (1.72) ****
PTCH1	***DIV65***<br>***NPC***	↑ (1.17) ****<br>↑ (1.25) ****	↑ (2.24) ****<br>↑ (1.00) ****	↑ (1.73) ****<br>↑ (1.15) ****
PTCHD1	***DIV65***<br>***NPC***	↑ (1.50) ****<br>↑ (2.14) ****	↑ (2.63) ****<br>↑ (2.33) ****	↑ (1.25) ****<br>↑ (1.55) ****
CEP76	***DIV65***<br>***NPC***	↓ (-0.46) ***<br>↓ (-0.42) **	↓ (-0.62) ****<br>↓ (-0.65) ****	↓ (-0.66) ****<br>↓ (-0.61) ****
CAV1	***DIV65***<br>***NPC***	↓ (-1.25) ****<br>↓ (-0.52) **	↓ (-1.33) ****<br>↓ (-0.42) **	↓ (-1.30) ****<br>↓ (-0.59) ***
APCDD1	***DIV65***<br>***NPC***	↓ (-0.56) ***<br>↓ (-0.94) ****	↓ (-0.36) *<br>↓ (-0.23) ****	-<br>↓ (-0.51) **
ROR1	***DIV65***<br>***NPC***	↓ (-0.72) ****<br>↓ (-0.84) ****	↓ (-0.76) ****<br>↓ (-0.72) ****	↓ (-0.44) ***<br>↓ (-0.47) ***

Table S2: Differentially expressed genes from the *WNT* and *SHH* signaling pathways after microarray on ZFN (Sigma) and patient-derived IVS10+16 NPCs and neurons (DIV65, in blue color) compared to the Sigma parental control. LogFC (fold changes) are shown for all significantly different genes with P<0.05. *P<0.05, **P<0.01, ***P<0.001 and ****P<0.0001. See also Figure 6.

Table S3: Differential expression of genes related to MAPT exon 10 splicing mediators due to IVS10+16 mutation. Related to Figure 6.

MAPT exon 10 splicing mediator GENES	Sigma IVS10+16 (logFC)	Patient 1 IVS10+16 (logFC)	Patient 2 IVS10+16 (logFC)
CELF3	↑ (0.36) *** NPC	↑ (0.27) ** NPC	↑ (0.32) ** NPC
CELF4	↓ (-0.52) *** NPC<br>↑ (0.56) *** wk5	↑ (0.55) *** NPC<br>↑ (1.25) **** wk5	↑ (1.03) **** NPC<br>↑ (0.89) **** wk5
NOVA1	↑ (0.64) **** NPC<br>↑ (1.33) **** wk5	↑ (0.90) **** NPC<br>↑ (1.92) **** wk5	-<br>↑ (1.76) **** wk5
SWAP70	-<br>↓ (-0.46) *** wk5	↑ (0.37) * NPC<br>↓ (-0.55) *** wk5	-<br>↓ (-0.42) ** wk5
SRSF3	↑ (0.27) ** wk5	-	↑ (0.38) **** wk5
SRSF1	↑ (0.39) **** wk5	-	↑ (0.62) **** wk5
SRSF4	↓ (-0.25) ** wk5	↓ (-0.30) *** wk5	-
SRSF6	↑ (0.78) **** wk5	↑ (0.85) **** wk5	↑ (1.26) **** wk5
DDX5	↑ (0.19) ** wk5	↑ (0.21) ** wk5	↑ (0.42) **** wk5
PTBP1	↓ (-0.58) **** NPC<br>↓ (-0.43) **** wk5	↓ (-0.34) **** wk5	↑ (0.15) * wk5
PRKACA	-<br>↑ (0.42) *** wk5	-<br>↑ (0.50) **** wk5	↑ (0.24) * NPC<br>↑ (0.30) ** wk5
DYRK1A	↑ (0.22) ** wk5	↑ (0.26) ** wk5	↑ (0.40) **** wk5
CLK2	↓ (-0.51) **** wk5	↓ (-0.30) ** wk5	-

Table S3: Differentially expressed genes from the *MAPT* exon 10 splicing machinery after microarray on ZFN (Sigma) and patient-derived IVS10+16 NPCs and neurons compared to the Sigma parental control. LogFC (fold changes) are shown for all significantly different genes with P<0.05. *P<0.05, **P<0.01, ***P<0.001 and ****P<0.0001. See also Figure 6.

Table S4: Differential expression of genes related to AD, PD and FTD due to IVS10+16 mutation. Related to Figure 6.

AD, PD and FTD related GENES	Sigma IVS10+16 (logFC)	Patient 1 IVS10+16 (logFC)	Patient 2 IVS10+16 (logFC)
APOE	(-1.50) **** wk5	(-1.48) **** wk5	(-1.73) **** wk5
BACE1	(0.35) *** wk5	(0.44) **** wk5	-
MAPT	(1.04) **** wk5	(1.37) **** wk5	(0.70) **** wk5
FUS	(0.24) *** wk5	-	(0.27) **** wk5
TARDBP	(0.22) * wk5	(0.28) ** wk5	(0.58) **** wk5
GRN	(-0.29) ** NPC	(-0.41) *** NPC	(-0.31) *** NPC<br>(-0.25) * wk5
CHCHD2	(-0.55) **** NPC<br>(-0.95) **** wk5	(-0.42) **** NPC<br>(-0.95) **** wk5	(-0.22) ** NPC<br>(-0.72) **** wk5
GAK	(0.47) ** wk5	(0.83) **** wk5	(0.56) *** wk5

Table S4: AD, PD and FTD-related differentially expressed genes after microarray on ZFN (Sigma) and patient-derived IVS10+16 NPCs and neurons compared to the Sigma parental control. LogFC (fold changes) are shown for all significantly different genes with P<0.05. *P<0.05, **P<0.01, ***P<0.001 and ****P<0.0001. See also Figure 6.

Table S5: Differential expression of genes related to calcium signaling due to P301S mutation. Related to Figure 7.

Significantly different genes potentially related with phenotypes	IVS10+16 / P301S DIV65 / DIV80 (logFC)	P values DIV65 / DIV80
CACNA2D3	↓ (-1.04) / (-0.67)	**** / ****
SLC8A1	↓ (-0.54)	**** (DIV65)
S100A16	↓ (-0.58) / ↓ (-0.60)	*** / ***
SMOC1	↓ (-0.74) / ↓ (-0.61)	** / *
CABYR	↑ (0.57)	** (DIV65)
S100A10	↑ (0.53) / ↑ (0.56)	* / *
NRXN3	↓ (-0.70) / ↓ (-0.65)	**** / ***
PRKCB	↓ (-0.38)	* (DIV65)

Table S5: List of differentially expressed genes after microarray analysis on P301S/IVS10+16 five to seven weeks after final plating and compared to its respective control (IVS10+16 +/+). Calcium signaling-related genes have been selected with a potential link to the observed phenotype. LogFC (fold changes) are shown. *P<0.05; **P<0.01; ***P<0.001; ****P<0.0001. See also Figure 7.

Supplemental experimental procedure

Human iPSC culture and differentiation into cortical neurons

Human iPSCs were cultured feeder-free and fed daily with fresh mTeSR™1 medium (Stem Cell Technologies). Cells were passaged with EDTA (Gibco) at confluency. Differentiation into neural progenitor cells (NPCs) and cortical neurons was performed using an adapted dual SMAD inhibition protocol (Kuijlaars et al., 2016, Shi et al., 2012). The only difference with the original protocol (Shi et al., 2012) implies the addition of BDNF, GDNF (both 10ng/ml) and cAMP (100μM) to the neural maintenance medium, leading to a mixture of both glutamatergic and GABAergic cortical neurons instead of purely glutamatergic neurons. For functional calcium experiments, neurons were co-cultured with human primary astrocytes (Kuijlaars et al., 2016) (ScienCell™) that were cultured and passaged per manufacturer's instructions (in human astrocyte medium, ScienCell™). All control and ZFN gene edited lines are deposited in EBiSC (https://cells.ebisc.org) as SIGi001-A (parental control, female), SIGi001-A-9 (IVS10+16 bi-allelic + P301S bi-allelic), SIGi001-A-12 (IVS10+16 bi-allelic) and SIGi001-A-13 (IVS10+16 mono-allelic). The iPSC lines from two female patients carrying the IVS10+16 (mono-allelic) mutation (patient 1 and patient 2) have been described (Sposito et al., 2015). Both patients were pre-symptomatic at the time of biopsy.

Seamless introduction of point mutations by gene editing using ZFN technology

Gene editing was performed by Sigma Aldrich on the control human iPSC line (cat#IPSC0028). Briefly, low passage number feeder free cultured hiPSCs were transfected with *in vitro* transcribed ZFN and single strand DNA oligo (ssODN) donor template by Amaxa nucleofection. The ZFN targets sequence CCT TCA CAC GTC CCA TGC GCC GTG CTG TGG CTT GAA TTA TTAG in intron 10 (underlined: ZFN binding sites). The 2 ssODNs used in this study were 102-nt long and contained the IVS10+16 SNP (C>T underlined) alone or in combination with the P301S point mutation (C>T in bold) (ssODN sequence= GTG GCT CAA AGG ATA ATA TCA AAC ACG TC**T** CGG GAG GCG GCA GTG TGA GTA CCT TCA CAT GTC CCA TGC GCC GTG CTG TGG CTT GAA TTA TTA GGA AGT GGT. ZFN activity was confirmed on genomic DNA from nucleofected cell pool in the mismatch cleavage assay. Cells were plated in pools and screened by Restriction Fragment Length Polymorphism (RFLP) using *Pci*I restriction enzyme for the presence of the IVS10+16 (C>T) point mutation and *Bsm*B1 for the presence of the p.P301S (C>T) point mutation. Selected pools were then single-cell plated and correctly edited clones identified by Sanger sequencing. Clonal populations containing either mono- or bi-allelic modifications for P301S alone or in combination with IVS10+16 were characterized for pluripotency (flow cytometry) and genomic stability (G banding).

RNA extraction and RTqPCR

Cells were lysed with RLT buffer (Qiagen) supplemented with 1% β-mercaptoethanol. RNA extraction was done using the RNeasy mini kit (Qiagen) followed by cDNA preparation using SuperScript® III (Life Technologies). For RTqPCR, the following Taqman assays to detect total MAPT and housekeeping genes were purchased at Life technologies total MAPT (Hs00902194_m1), PGK1 (Hs99999906_m1), HMBS (Hs00609297_m1) and PPIB (Hs00168719_m1). SOX3, CDK6, CDK4, WLS, ROR1, PITCH, NEUROD1, EOMES, DLX5 and APCDD1 Taqman assays were purchased at IDT. The assays to specifically detect the MAPT variants with or without exon 10 were custom designed and ordered at IDT: TaqMan assay MAPT_ 3R_exon 9_11 (Fwd primer: GCT CCA CTG AGA ACC TGA AG, Rev primer: CCT AAT GAG CCA CAC TTG GA, Probe 56-FAM/AG ACT ATT T/Zen/G CAC CTT CCC GCC TC/3IABkFQ) and TaqMan assay MAPT_4R_Exon 9_10 (Fwd primer: GCT CCA CTG AGA ACC TGA AG, Rev: TTG AGC CAC ACT TGG ACT G, Probe 56-FAM/AA TTA TCT G/Zen/C ACC TTC CCG CCT CC/3IABkFQ. Normalization was done using the geNorm method ((Vandesompele et al., 2002)) using reference genes (B2M, HMBS, PGK1, PPIB, TFRC). A normalization factor was computed as the geometric averaging of the gene expression values of the most stable reference genes. The gene expression values were divided by this normalization factor. A second normalization was performed to the parental control at NPC stage.

Microarray analysis

For microarray analysis, RNA was extracted with the RNeasy 96 kit (cat# 74181 Qiagen). All microarray-related steps for target preparation, including the amplification of total RNA and labeling, were carried out as described in the GeneChip®3' IVT Express Kit User Manual (Affymetrix 2004). Biotin-labeled target samples were hybridized to the GeneChip® HG-U219 containing probes for over 18k genes. Target hybridization was processed on the GeneTitan® Instrument according to the instructions provided in the User Guide for Expression Array Plates (P/N 702933). Images were analyzed using the GeneChip® Command Console Software (AGCC) (Affymetrix). All microarray data were processed using the statistical computing R-program (R version 3.1.1 (Team, 2015)) as well as Bioconductor tools (Gentleman et al., 2004). The gene expression values were normalized using Robust Multi-array Average (RMA)

(Irizarry et al., 2003). Grouping of the individual probes into gene-specific probe sets was performed based on Entrez Gene using the metadata package hgu219hsentrezg (version 20.0.0) (Dai et al., 2005).

Single cell data analysis

Single-nucleus RNA sequencing data originating from the cerebral cortex of a postmortem brain (Lake et al., 2016) were downloaded from the Sequence Read Archive (accession SRP052546). Transcript-level quantification was performed with kallisto (Bray et al., 2016) using Ensembl release 89 as a reference transcriptome, and further processed using the R Bioconductor package scater (McCarthy et al., 2017). Gene-level summarization was performed by grouping all transcripts in a given gene, except for the MAPT gene, where grouping was performed using transcripts either including or excluding exon 10.

Live Cell Calcium Imaging

Cells were loaded with 1 µM Fluo-4-AM (Thermo Fisher Scientific) in D-PBS containing calcium and magnesium (cat# D8662; Sigma) with addition of 10mM glucose. Cultures were incubated at 37°C and 5% CO2 for 30 minutes and then imaged with an inverted confocal laser scanning microscope (Axiovert 100M Carl Zeiss, combined with Zeiss LSM510 software) using a Plan-NEOFLUAR 20x objective lens (NA 0.50). 250 frames (61 frames per minute) were recorded per well followed by 30 µM glutamate addition (50 frames) to distinguish neurons from non-neuronal cells (Pickering et al., 2008). Traces of non-neuronal cells, showing only a transient increase in fluorescence intensity upon glutamate addition, were discarded. A custom-made MATLAB script (based on (Cornelissen et al., 2013)) was used to analyze live cell calcium traces and to derive various parameters reflecting characteristics of neuronal activity. Fluorescence traces were normalized to the initial fluorescence intensity (F/F0) and the average calcium burst frequency was calculated for the active cells. Active cells were defined as cells showing at least one peak (i.e. calcium burst) in the fluorescence signal.

Western Blot

Cells were washed gently with PBS and lysed in RIPA buffer (Gibco) supplemented with protease and phosphatase inhibitors (HALT®; Invitrogen). To detect separate 3R and 4R tau isoforms, some of the samples were dephosphorylated with lambda phosphatase (New England Biolabs) according to the manufacturer's protocol after acetone precipitation, to obtain a maximum yield of proteins. Protein was loaded on either 4-12% or 10% Criterion Bis-Tris gels (Biorad) and after SDS PAGE, gels were blotted on nitrocellulose and blocked for 1 hour at room temperature (RT) in TBS-0.1% Tween-20 supplemented with 5% milk. The primary rabbit anti-total tau (1□g/ml; DAKO #A0024), mouse anti-RD4 (1/1000; Millipore, 50µg protein loaded), mouse anti-RD3 (1/2000; Millipore, 10 µg protein loaded) antibodies was were incubated overnight at 4^{0}C in blocking buffer. A tau ladder (rPeptide) with all 6 tau isoforms was included to distinguish between 3R and 4R isoforms after dephosphorylation. Detection was done with HRP-labeled secondary antibodies (GE Healthcare) and the West Dura® or West Femto® enhanced chemiluminescence kit (Pierce, Thermo Scientific). Blots were stripped and reprobed with mouse anti-β-actin (1/5000; Sigma) and anti-β3 tubulin (1/1000; Covance) as loading controls.

Preparation of K18 fibrils

Monomeric tau K18-P301L or K18-wild type (40 µM, N and C-terminal myc-tagged) was mixed with 40 µM of heparin, 2mM DTT and 100 mM sodium acetate buffer (pH 7.0) and incubated at 37°C for 48 - 72 hours. Afterwards, the mix was centrifuged (100.000g, 1 hour at 4°C). The supernatant was discarded and the pellet was resuspended in the same final volume of sodium acetate buffer. K18 was freshly sonicated before use (60 cycles of 2 second pulses).

Transduction of NPCs with AAV6-4R tau of AAV6-GFP

Control cortical neural progenitor cells in MW6 plates were transduced with AAV6-syn1-TAU-2N4R (produced by Sebastian Kügler, Dept. of Neurology, University Medicine, Goettingen, Germany) or AAV6-syn1-GFP (produced by SIRION Biotech) at an MOI of 150 or 300 and with final plating of the cells 24 hours later in PLO/laminin coated MW96 plates.

Generation of Tau BRET reporter cell line and Nano BRET™ Nano-Glo® assay

Synthetic fragments of Nano-Luc, an ATP-independent luciferase, or HaLo tag from Promega were cloned in-frame to the C-terminal side of a K18 Tau fragment existing solely of the four MT binding domains with P301L mutation. Both were cloned into the pcDNA5/TO or pcDNA4/TO mammalian expression vector respectively (Invitrogen, Carlsbad, CA, USA). In these vectors, receptor expression is under the control of a CMV promoter and two tetracycline operators, which confers tetracycline-inducible expression on the insert. Hek293 cells were co-transfected

with K18P301L-NanoLuc-pcDNA5/TO, K18P301L-HaloTag-pcDNA4/TO, and pcDNA6/TR expressing the Tet repressor (Invitrogen, Carlsbad, CA, USA) using lipofectAMINE 2000 (Gibo-BRL, Eggenstein, Germany) according to the recommendations of the supplier. Monoclonal cell lines were isolated under Zeocin (200 mg/ml), Hygromycin (200 µg/ml), and Blasticidin (5 mg/ml) in DMEM medium supplemented with 10% heat inactivated fetal calf serum and antibiotics (Life Technologies, Gaithersburg, MD, USA). The same medium was used for cell culturing. Zeocin, Hygromycin, and Blasticidin were left out at least 1 day before any assay. The selected monoclonal line used in the assay showed expression of both constructs in the absence of tetracyclin, therefore the Tet repressor was not blocked before the BRET assay. Wild type K18 fibrils were added to the cell line and incubated for 72 hours, followed by addition of NanoBRET™ Nano-Glo® Substrate and measurement of the signal (donor emission at 460 nm and acceptor emission at 618 nm) within 10 minutes after substrate addition.

Tau insoluble Sarkosyl extraction

Cell pellets were thawed on ice and lysed in RIPA buffer (Sigma # R0278) (Merck, Sigma) supplemented with cOmplete™ (EDTA free) and PhosSTOP™ (Roche) as recommended by the manufacturer. Samples were homogenized on a rocker (IKA Loopster digital) at 4°C. Equal amounts of protein (50-100µg) were aliquoted after BCA protein quantification (Sigma BCA1-1KT) and supplemented with a 20% N-Lauroylsarcosine (Sigma # L9150) solution in RIPA to bring N-Lauroylsarcosine at a final concentration of 1% (w/v). Samples were homogenized on a rocker for 1 hour at room temperature. Samples were then centrifuged at 100,000g for 1 hour at room temperature in a Beckman coulter ultima ultracentrifuge using a TLA-100 rotor and corresponding centrifugation tubes (Beckman, coulter #342303). Supernatants (Soluble fraction) were separated from pellets (insoluble fraction) and kept at -80°C until further analysis. For western Blots analysis, supernatant or pellets were supplemented with LDS (ThermoFisher, Life Technologies, # NP0005) and sample reducing agent (ThermoFisher, Life Technologies, # NP0004) and loaded onto Novex Nupage gels as recommended by the manufacturer. Human Tau monoclonal antibody HT7 (ThermoFischer, MN1000) and actin (Merck, Millipore, # MAB1501) antibodies were used for detection. As a positive control, QBI cells overexpressing 2N4R-TauP301L and seeded with K18P301L were used. The cells were generated by transient transfection with 2N4R-TauP301L-pcDNA4 plasmid in optiMEM using Fugene6 transfection reagent (Promega) according to manufacturer's recommendation. After 24 hours, cells were then either seeded or not with sonicated K18P301L fibrils in a Bioporter (BP609504) sodium acetate buffer (0.1M, pH7) solution and incubated for 3 hours. Cell medium was then replaced with fresh optiMEM supplemented with 20% Foetal bovine serum and 2% Penicillin/streptomycin (Life Technologies) and then cultured for 48 hours prior lysis in RIPA buffer with protease and phosphatase inhibitors as described above.

Table S6: Antibody information. Related to materials and methods.

Antibody	Provider	Catalogue number
rabbit anti tau	DAKO	A0024
mouse anti-actin	Millipore	MAB1501
rabbit anti-VGLUT2	Synaptic systems	135403
rabbit anti-OCT4	Invitrogen	A13998
mouse anti- ISL1	Thermo Fisher	MA5-15516
mouse anti-RD4	Millipore	05-804
mouse anti RD3	Millipore	05-803
mouse anti actin	Millipore	MAB1501
mouse anti-NANOG	Millipore	MABD24
mouse and rabbit anti-TUBB3	Covance (BioLegend)	MMS-435P<br>PRB-435P<br>MRB-435P
rabbit anti-TTF1	Abcam	ab76013
rabbit anti-TBR1	Abcam	ab31940
rat anti-CTIP2	Abcam	ab18465
Click-iT® Plus EdU Alexa Fluor® 594 Imaging kit	Invitrogen	C10639
TUNEL (Roche) kit	Roche/Sigma	11 684 795 910

Supplemental References

J. Kuijlaars, T. Oyelami, A. Diels, J. Rohrbacher, S. Versweyveld, G. Meneghello, M. Tuefferd, P. Verstraelen, J. R. Detrez, M. Verschuuren, et al. Sustained synchronized neuronal network activity in a human astrocyte co-culture system. *Sci Rep,* 6 (2016)**,** 36529.

Y. Shi, P. Kirwan & F. J. Livesey Directed differentiation of human pluripotent stem cells to cerebral cortex neurons and neural networks. *Nat Protoc,* 7 (2012)**,** 1836-46.

T. Sposito, E. Preza, C. J. Mahoney, N. Seto-Salvia, N. S. Ryan, H. R. Morris, C. Arber, M. J. Devine, H. Houlden, T. T. Warner, et al. Developmental regulation of tau splicing is disrupted in stem cell-derived neurons from frontotemporal dementia patients with the 10 + 16 splice-site mutation in MAPT. *Hum Mol Genet,* 24 (2015)**,** 5260-9.

J. Vandesompele, K. De Preter, F. Pattyn, B. Poppe, N. Van Roy, A. De Paepe & F. Speleman Accurate normalization of real-time quantitative RT-PCR data by geometric averaging of multiple internal control genes. *Genome Biol,* 3 (2002)**,** RESEARCH0034.

R. C. Team A language and environment for statistical computing. R Foundation for Statistical Computing, Vienna, Austria. (2015).

R. C. Gentleman, V. J. Carey, D. M. Bates, B. Bolstad, M. Dettling, S. Dudoit, B. Ellis, L. Gautier, Y. Ge, J. Gentry, et al. Bioconductor: open software development for computational biology and bioinformatics. *Genome Biol,* 5 (2004)**,** R80.

R. A. Irizarry, B. Hobbs, F. Collin, Y. D. Beazer-Barclay, K. J. Antonellis, U. Scherf & T. P. Speed Exploration, normalization, and summaries of high density oligonucleotide array probe level data. *Biostatistics,* 4 (2003)**,** 249-64.

M. Dai, P. Wang, A. D. Boyd, G. Kostov, B. Athey, E. G. Jones, W. E. Bunney, R. M. Myers, T. P. Speed, H. Akil, et al. Evolving gene/transcript definitions significantly alter the interpretation of GeneChip data. *Nucleic Acids Res,* 33 (2005)**,** e175.

B. B. Lake, R. Ai, G. E. Kaeser, N. S. Salathia, Y. C. Yung, R. Liu, A. Wildberg, D. Gao, H. L. Fung, S. Chen, et al. Neuronal subtypes and diversity revealed by single-nucleus RNA sequencing of the human brain. *Science,* 352 (2016)**,** 1586-90.

N. L. Bray, H. Pimentel, P. Melsted & L. Pachter Near-optimal probabilistic RNA-seq quantification. *Nat Biotechnol,* 34 (2016)**,** 525-7.

D. J. Mccarthy, K. R. Campbell, A. T. Lun & Q. F. Wills Scater: pre-processing, quality control, normalization and visualization of single-cell RNA-seq data in R. *Bioinformatics,* 33 (2017)**,** 1179-1186.

M. Pickering, B. W. Pickering, K. J. Murphy & J. J. O'connor Discrimination of cell types in mixed cortical culture using calcium imaging: a comparison to immunocytochemical labeling. *J Neurosci Methods,* 173 (2008)**,** 27-33.

F. Cornelissen, P. Verstraelen, T. Verbeke, I. Pintelon, J. P. Timmermans, R. Nuydens & T. Meert Quantitation of chronic and acute treatment effects on neuronal network activity using image and signal analysis: toward a high-content assay. *J Biomol Screen,* 18 (2013)**,** 807-19.
